# (Cr,Mn,Fe,Ni,Zn) High-Entropy Oxides as Electrocatalysts for Green Hydrogen Production via Anion Exchange Membrane Water Electrolysis

**DOI:** 10.3390/nano16161034

**Published:** 2026-08-20

**Authors:** Sabrina Campagna Zignani, Marta Fazio, Mariarosaria Pascale, Chiara Alessandrello, Claudia Triolo, Maria Grazia Musolino, Saveria Santangelo

**Affiliations:** 1 Institute of Advanced Technologies for Energy (ITAE), The National Research Council (CNR), Salita S. Lucia Sopra Contesse 5, 98126 Messina, Italy; fazio@itae.cnr.it (M.F.); pascale@itae.cnr.it (M.P.); 2 Department of Civil, Energy, Environmental and Materials Engineering (DICEAM), Mediterranean University of Reggio Calabria, Via R. Zehender, Loc. Feo di Vito, 89122 Reggio Calabria, Italy; c.alessandrello@unirc.it (C.A.); claudia.triolo@unirc.it (C.T.); 3 National Reference Center for Electrochemical Energy Storage (GISEL), National Interuniversity Consortium for the Science and Technology of Materials (INSTM), 50121 Florence, Italy

**Keywords:** co-free high-entropy oxides, sol-gel method, anion-exchange membrane water electrolysis, oxygen evolution reaction, hydrogen evolution reaction, membrane electrode assemblies

## Abstract

The development of sustainable and low-cost electrocatalysts based on Earth-abundant elements is essential for the large-scale deployment of green hydrogen production via anion exchange membrane water electrolysis (AEMWE). Herein, we demonstrate the feasibility of cobalt-free high-entropy oxide (HEO) electrodes for AEMWE through a set of spinel oxides based on equimolar Cr, Mn, Fe, Ni, and Zn. The HEOs were synthesized by a scalable sol-gel route followed by calcination at different temperatures (400–800 °C). The pristine oxides were employed as oxygen evolution reaction catalysts, whereas their H_2_/Ar-reduced counterparts were used as hydrogen evolution reaction catalysts in symmetric membrane electrode assemblies (MEAs). Comprehensive physicochemical characterization combined with electrochemical testing revealed that the phase purity of the anodic catalyst mainly correlates with both the maximum current density and the polarization resistance of the electrolyzer. In contrast, a correlation is observed between the physicochemical features of the reduced cathodic catalyst, the *iR*-free potential and the polarization resistance after prolonged operation. The best-performing Co-free MEA achieved a current density of 0.51 A cm^−2^ at 2.2 V and exhibited stable operation for hundreds of hours under alkaline electrolysis conditions. Although the complete replacement of cobalt results in lower activity than previously reported Co-containing HEOs, the present work establishes a viable design strategy for fully Co-free electrocatalysts and highlights the critical balance between catalytic performance, long-term stability, and material sustainability in future AEMWE technologies.

## 1. Introduction

The global energy sector currently faces challenges arising from escalating demand and pressing environmental concerns. The use of exhaustible fossil fuels as a primary source of energy has contributed to their depletion and to a significant rise in carbon dioxide and other greenhouse gases (GHGs) in the atmosphere, which are responsible for global warming and climate change. Therefore, the development of renewable and sustainable energy sources has become a vital priority to achieve the goal of net zero emissions by 2050 [1,2]. Hydrogen, as a clean and versatile energy carrier, is considered a viable alternative to traditional fossil fuels, making it a potential key enabler for the energy transition. H_2_ has an exceptionally high gravimetric energy density (~140 MJ/kg)—much higher than that of traditional fuels—and offers the benefits of being abundant, non-toxic and environmentally friendly, yielding only water as a by-product upon combustion [3]. However, its sustainable production is heavily dependent on the energy source and technology used. Currently, H_2_ is mainly produced worldwide via methane steam reforming and coal gasification, due to their technological maturity and relatively low cost [4]. Unfortunately, these methods consume fossil fuels, produce low-purity hydrogen, and release large amounts of carbon emissions when not integrated with carbon capture, utilization, and storage units. Electrochemical water electrolysis (WE), especially when powered by renewable energy sources (e.g., solar and wind), provides a reliable, sustainable, and highly efficient route to produce green hydrogen on a large-scale. Thanks to its high purity and zero GHG emissions, green H_2_ represents a promising solution for the full de-carbonization of hard-to-abate sectors such as heavy industry, heating, long-distance transport, aviation, and shipping [5].

The WE process (H_2_O_(l)_ ⟶ H_2(g)_ + 0.5 O_2(g)_) consists of two half-cell reactions that occur simultaneously at the electrode–electrolyte interface: the cathodic hydrogen evolution reaction (HER) and the anodic oxygen evolution reaction (OER). The overall reaction mechanism involves multiple proton–electron transfer steps (2e^−^ for HER, 4e^−^ for OER) and intermediate species whose stability is strongly affected by the nature of the electrolyte used in the electrolyzer. WE is an energy-intensive reaction and requires a theoretical thermodynamic potential of 1.23 V (at 25 °C, 1 atm). In practice, a considerable higher cell voltage (1.8–2.0 V) is required to overcome the intrinsic energy barriers associated with sluggish pathways of both the HER and OER reactions, thereby generating an appreciable current density [6]. This overpotential stems from complex electron and ion transport pathways, leading to energy losses and reduced electrolyzer efficiency, which ultimately increases operating costs. Therefore, the development of efficient electrocatalysts for both the HER and OER is essential to accelerate the reaction kinetics, reduce the overpotential, and make WE technology energetically favorable. Platinum group metal (PGM)-based electrocatalysts, such as Pt for the HER and IrO_2_/RuO_2_ for the OER, exhibit excellent catalytic performance for overall WE; however, their high cost and scarcity severely impede their large-scale commercialization [7].

Among various WE technologies, anion exchange membrane water electrolysis (AEMWE) stands out as an efficient, cost-effective and low-temperature option for green hydrogen production [8,9]. AEMWE combines the advantages of both liquid alkaline water electrolysis (AWE) and proton exchange membrane water electrolysis (PEMWE). It utilizes the milder alkaline environment of AWE, allowing the use of non-PGM electrocatalysts, while maintaining the high efficiency, high current density, high hydrogen purity, and compact cell configuration typical of PEMWE. Despite these advantages, the widespread deployment of AEMWE still faces considerable kinetic challenges at the electrodes. Consequently, extensive research efforts are underway to design and develop highly active, durable, and eco-friendly electrocatalysts made from Earth-abundant raw materials.

Recently multi-metallic nanomaterials based on the high-entropy concept have opened a new paradigm for catalyst design. High-entropy materials (HEMs) possess a single-phase solid solution structure stabilized in a multi-component system (generally, containing five or more elements in equimolar or near-equimolar ratio) by high configurational entropy [10]. Unlike conventional low- or medium-entropy materials, HEMs—including high-entropy alloys, high-entropy oxides, high-entropy nitride, high-entropy fluorides, high-entropy borides, high-entropy carbides, high-entropy sulfide, high-entropy layered double hydroxides, and high-entropy phosphides—exhibit excellent thermodynamic stability and enhanced catalytic performance. This is owing to their pronounced lattice distortion, sluggish diffusion and synergistic electronic interactions (known as the “cocktail effect”) among the constituent species [11,12,13,14].

Very recently, it has been demonstrated that nanostructured (Cr,Mn,Fe,Co,Ni)-based high-entropy oxides (HEOs) have great potential not only as OER electrocatalysts in alkaline environment, but also for simplifying the electrode manufacturing process, thereby reducing the overall costs of AEMWE systems, while simultaneously delivering performance levels suitable for industrial application [15]. Moreover, Co-containing nanomaterials have emerged as highly efficient cathode candidates for AEMWE, demonstrating not only superior kinetics for the HER, but also robust chemical stability in harsh alkaline environments [16,17]. However, the reliance on cobalt poses significant sustainability bottlenecks, as cobalt has been officially designated as a critical raw material (CRM) by both the European Union and the United States due to its severe supply chain vulnerability [18]. Global cobalt extraction is characterized by extreme geographic concentration; the world’s supply mainly originates from the Democratic Republic of the Congo, a country associated with heightened geopolitical risks. Concurrently, the massive demand for cobalt from the rapidly expanding rechargeable battery market exposes world industries to supply disruptions, export restrictions, and price volatility, driving up material costs [19,20]. Consequently, engineering “cobalt-free” electrocatalysts by utilizing non-critical, eco-friendly, and Earth-abundant transition metals has emerged as a major prerequisite for ensuring truly sustainable and socio-economically viable green hydrogen value chains.

The incorporation of redox-inactive Zn^2+^ ions into spinel-type transition metal oxides modifies the local charge density and the coordination environment around the transition metal active sites [21]. Zn doping optimizes the electronic structure of cobalt–nickel sulfide, enhancing its conductivity and charge transfer capability [22].

Building upon this imperative, this study examines the feasibility of extending the previously proposed electrode design strategy to a “second-generation” of HEOs entirely free of socio-economically critical or sensitive elements. Specifically, a set of Co-free (Cr, Mn, Fe, Ni, Zn) HEOs is synthesized via a simple and scalable method and its performance is comprehensively evaluated within a zero-gap AEMWE cell. The study goes beyond merely presenting the results of elemental substitution; it highlights for the scientific community the structural, chemical, and operational challenges involved in dealing with zinc-containing high-entropy oxides and, more generally, zinc-doped catalysts.

## 2. Experimental Section

### 2.1. Preparation and Physicochemical Characterization of Electrocatalysts

As-purchased (from Merck) reactants were utilized to prepare the electrocatalysts. Details on the preparation procedure [23] can be found in the Supporting Information (SI). The sol-gel (SG) method (Figure S1), easily scalable and suitable for large-scale production, was used, followed by calcination in air for 2 h at the selected temperature (*T*_C_: 400, 500, 600, or 800 °C) and rapid cooling down to room temperature (RT) out of the furnace to generate defects on the oxide surface [23,24], enhance porosity and oxygen deficiency [25], and kinetically trap the metastable phase of the material [26]. The oxides were coded as HEO-*T*_C_. A portion of each was evaluated as anodic material, and the remaining portion was further processed in 5% H_2_/Ar atmosphere for 0.5 h to obtain the cathodic material, hereafter coded as HEO-*T*_C_/R. Further details can be found in the SI.

The physicochemical properties of all electrode materials were investigated by scanning electron microscopy-energy dispersive X-ray (SEM-EDX), X-ray diffraction (XRD), and X-ray photoelectron spectroscopy (XPS). Micro-Raman spectroscopy (MRS) analysis was further carried out on the materials in the oxidic form. A SEM Thermo Fisher FEG-UHR microscope (2–15 kV) with FIB and STEM detectors provided information on morphology and elemental composition via EDX. A Bruker D2 diffractometer (Cu Kα, λ = 0.1541 nm) was used identify the phases formed; diffractograms were refined via the Rietveld method (Maud 2.992). Surface composition and chemical states were studied by XPS (PHI 5800-01, Al Kα 1486.6 eV, 300 W) using PHI MultiPak 6.1 and the PHI Handbook of XPS. The spatial homogeneity of the oxide in terms of phase composition was assessed via MRS (NTEGRA Spectra SPM NT-MDT, 532 nm laser, 250 µW, RT) measuring Raman scattering from several random positions in each sample.

### 2.2. Membrane Electrode Assembly and Electrochemical Studies

Catalytic inks were prepared by dispersing the catalyst powder together with the anionic ionomer (Fumatech^®^ ION FAA-3, 10 wt% solution) in ethanol. The resulting suspension was sonicated for 30 min to ensure homogeneous dispersion before being deposited by spray coating using an airbrush (Figure S3a). The HEO-based anode ink was sprayed onto nickel felt (Bekaert, Figure S3b), whereas the HEO/R-based cathode ink was deposited onto carbon paper (SIGRACET 39BB, Figure S3c). A commercial 50 μm Fumatech^®^ FAA3-50 membrane served as the solid polymer electrolyte (Figure S3d). Before assembly, the membrane was activated by immersion in a 3 M NaCl solution for 72 h. Membrane electrode assemblies (MEAs, Figure S3e) were fabricated through a cold-pressing procedure to avoid possible membrane degradation during assembly. Prior to the pressing step, both the membrane and the electrodes were immersed in an OH^−^-containing solution for 24 h to achieve complete ion exchange.

The AEMWE cell (Figure S3g) was assembled using nickel end plates (Bekaert) equipped with serpentine flow fields, providing an active area of 5 cm^2^. Teflon^®^ gaskets were employed to ensure proper sealing, while the cell components were compressed at a torque of 2.5 Nm per tie rod to guarantee intimate contact between the different layers. During operation, the anode was continuously supplied with 1 M KOH at a flow rate of 5 mL min^−1^ using a peristaltic pump. All electrochemical measurements were carried out under atmospheric pressure. The cell temperature was maintained at 50 °C by means of external heating mats, as this operating temperature represents an effective compromise between membrane durability and ionic conductivity [27].

To minimize degradation of the carbon paper, the maximum cell voltage was limited to 2.2 V. The electrochemical performance of each MEA was evaluated by recording galvanostatic polarization curves (cell voltage versus current density), conducting durability tests (cell voltage as a function of time), and performing electrochemical impedance spectroscopy (EIS) measurements. Galvanostatic polarization and chronopotentiometric experiments were carried out using a Keithley power supply (Tektronix) coupled with a PGSTAT Autolab 302 Potentiostat/Galvanostat equipped with a 20 A booster (Metrohm). EIS measurements were performed under potentiostatic conditions using a Frequency Response Analyzer (FRA) over the frequency range of 1 MHz to 10 mHz. A single-sine AC perturbation with an amplitude of 0.01 V rms was applied, and ten measurement points were collected per frequency decade.

## 3. Results and Discussion

### 3.1. Physicochemical Characterization

**Figure 1:**
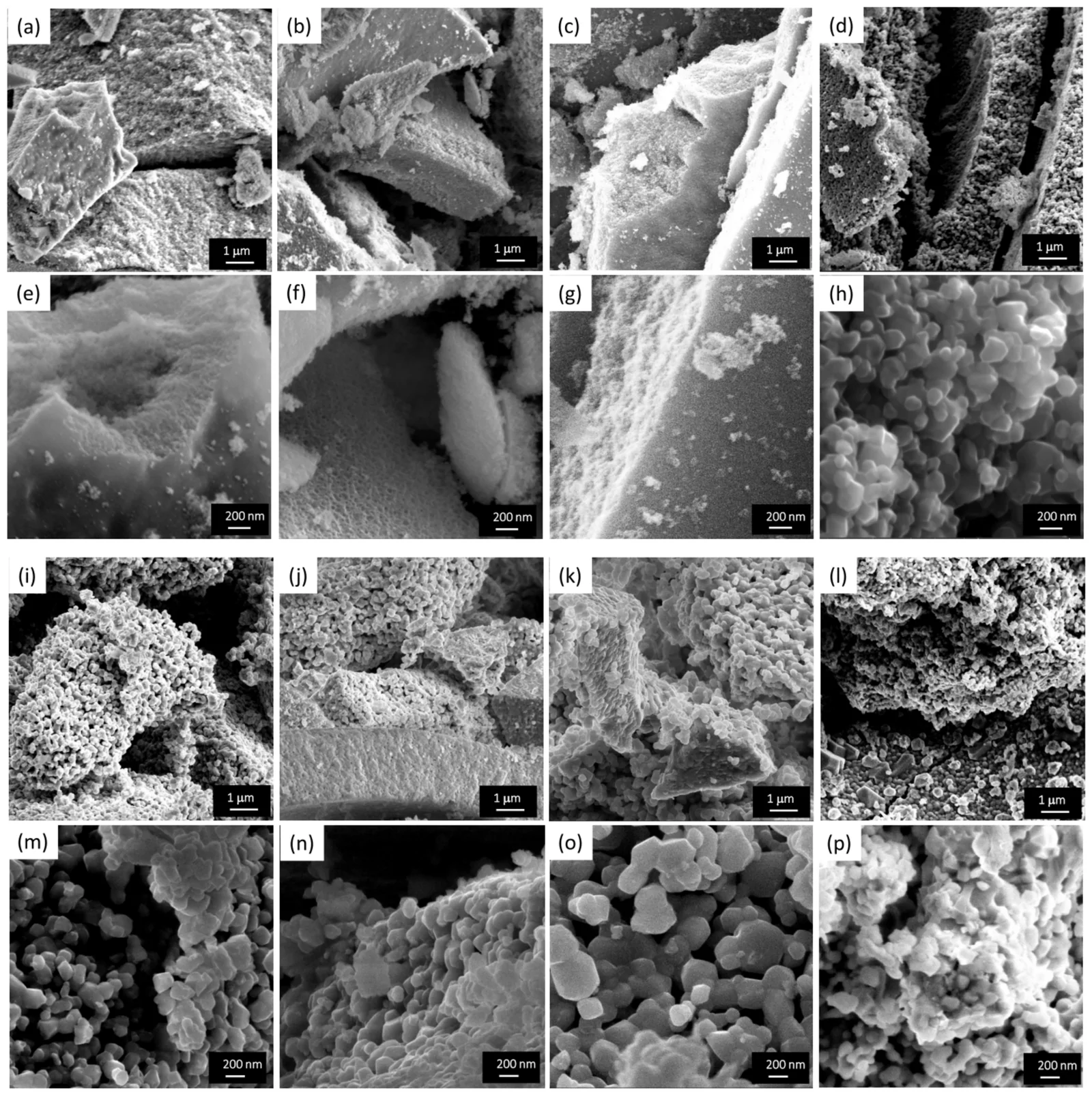
Electrocatalyst morphology as resulting from SEM analysis. The micrographs shown refer to samples (**a**,**e**) HEO-400, (**b**,**f**) HEO-500, (**c**,**g**) HEO-600, (**d**,**h**) HEO-800, (**i**,**m**) HEO-400/R, (**j**,**n**) HEO-500/R, (**k**,**o**) HEO-600/R and (**l**,**p**) HEO-800/R.

The electrocatalyst morphology is investigated by SEM, with representative micrographs shown in Figure 1. The typical morphology of the oxides produced by SG [23,28] is observed (Figure 1a–h): HEOs are made up of agglomerates of oxide nanoparticles (NPs) with distributed sizes. An increase in *T*_C_ produces evident sintering effects [29]; as a result, the average size of the oxide NPs increases from around tens of nm at 400 °C (Figure 1e) to hundreds of nm at 800 °C (Figure 1h). After exposure to H_2_/Ar atmosphere, the morphology of the reduced oxides does not change substantially (Figure 1g–p), in line with previous results [15]. A lower degree of agglomeration is observed along with a considerable increase in the average particle size, due to sintering effects, which become more marked as the difference between the calcination and reduction temperatures of the oxides widens.

Elemental analysis by SEM/EDX shows that at all *T*_C_, nearly equimolar multi-metallic oxides are formed (Figure S4a–d), in which oxygen and transition metals (TMs) have a spatially uniform distribution (Figure S5). The treatment in reducing atmosphere does not alter the molar fraction of the metals to any appreciable extent (Figure S4e–g) except in the case of sample HEO-800/R, in which a significant decrease in the molar fraction of zinc is observed (Figure S4h) compared to the unreduced oxide (Figure S4d and Table S1). This hints at the possible occurrence of a redistribution of this element beyond the region probed by the SEM/EDX and/or its leaching as reported in the literature for zinc ferrite [30].

**Figure 2:**
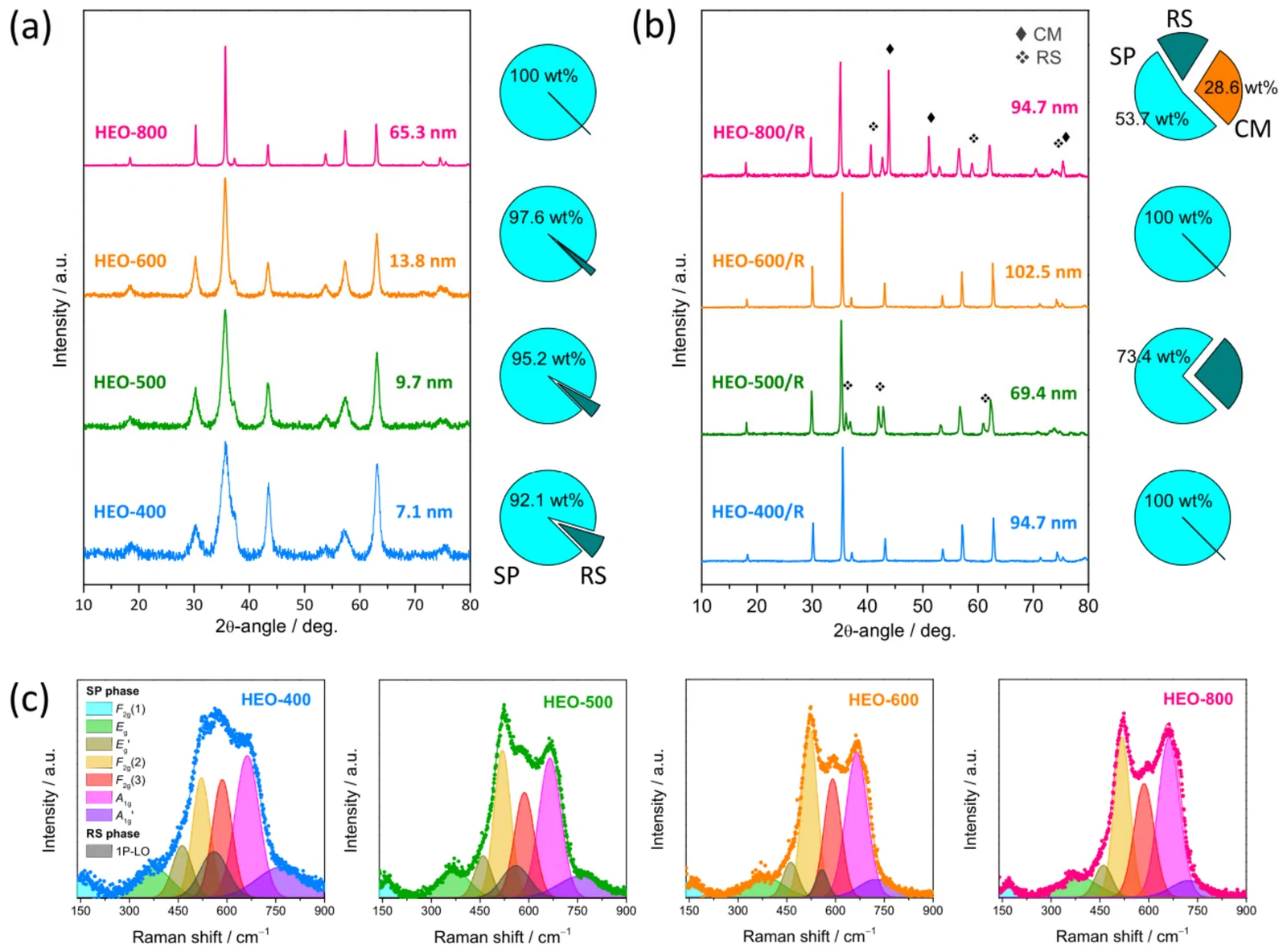
Results of (**a**,**b**) XRD and (**c**) MRS analyses on (**a**,**c**) OER and (**b**) HER electrocatalyst. The main results of Rietveld refinements to XRD data (average crystal size and phase composition) are also reported.

The phase composition of the electrocatalysts is investigated by XRD. Figure 2a,b displays the diffractograms of the as-calcined and reduced oxides, respectively, and their phase composition, as determined from Rietveld refinements (Figures S6 and S7, Tables S2 and S3). A pure single spinel (SP) oxide phase is obtained only by calcination at 800 °C (Figure 2a). At lower *T*_C_, part of metals segregates into a secondary phase with rock-salt (RS) structure. The relative amount of this phase decreases monotonically with *T*_C_ (Figure S8a), while the average of the oxide crystallites (*d*_HEO_) undergoes a rapid increase (Figure S8b), as already observed for HEOs prepared by a different method [25].

Very sharp diffraction peaks are detected in all reduced oxides (Figure 2b), as expected. The higher the reduction temperature (*T*_R_) compared to the temperature experienced during calcination, the larger the increase in the average crystallite size (*d*_HEO/R_) compared to the pristine oxides (Figure S8c). In all XRD patterns, reflection peaks from the SP lattice are still detectable, confirming the structural integrity of the bulk phase. The formation of a face-centered cubic (fcc) metallic phase is clearly observed only in HEO-800/R, where an MnO phase with RS structure also forms. In sample HEO-500/R, the relative amount of the RS phase increases significantly (from 4.8 to 26.6 wt%), revealing the partial reduction of the average oxidation state of the metal cations from +3 to +2. In HEO-400/R and HEO-600/R, the lack of additional diffraction signals from cubic metal/alloy (CM) and the disappearance of reflections from RS impurities could be indicative of the formation of alloy/metal domains that are not adequately resolved by bulk XRD and/or of the nanoscale elemental migration and segregation, as observed in HEO-based lithium ion battery anodes after prolonged cycling [31].

The spatial homogeneity of the oxide phase(s) is evaluated by measuring Raman scattering from several randomly selected locations on each sample (Figure S9). A comparison of the spectral profiles recorded for samples HEO-500 (Figure S9b), HEO-600 (Figure S9c), and HEO-800 (Figure S9d), reveals no significant differences in peak positions and relative intensities indicating that the formed phase(s) is (are) spatially uniform over the region probed by MRS (<0.6 μm^2^, under the present conditions). On the contrary, the slight differences in the spectral profiles recorded for HEO-400 (Figure S9a) suggest that the larger amount of RS impurities leads to non-homogeneity.

The five normal modes (*A*_1g_ + *E*_g_ + 3*F*_2g_) peculiar to the spinel-oxide lattice ($Fd\bar{3}m$ space group) [32,33,34] are identified by fitting the average spectra to Gaussian bands (Figure 2c). Inversion-induced modes also contribute to the Raman intensity in all oxides [32,33,34], whereas the one-phonon longitudinal optical (1P-LO) of the defect-rich [35,36,37] cubic RS-structured component of the oxide ($Fm\bar{3}m$ space group), detected for *T*_C_ ≤ 600 °C, vanishes at 800 °C, in full agreement with the outcomes of the Rietveld analysis. Furthermore, the inversion-bands progressively weaken with increasing *T*_C_, consistent with the *ideal* evolution of the cation distribution (CD) in the SP lattice [38,39] (Figure S10). The *T*_C_-driven structural evolution is accompanied by a change in configurational entropy ($∆S_{config}$ in Table S4), as roughly estimated based on the literature [40], and in the occupancy of the catalytically important 16*d* sites. The qualitative picture that emerges is that as *T*_C_ increases, the occupancy of 16*d* sites by highly active Ni^2+^ cations increases at the expense of catalytically inert Zn^2+^ cations (Figure S10). However, to a lesser extent, the occupancy of octahedral sites by Cr^3+^ and Mn^3+^ also increases at the expense of Fe^3+^ cations, which possess a lesser octahedral site preference energy [39]. As a result, the occupancy of *e_g_* orbitals at octahedral sites varies as well. The optimal *e_g_* filling (≈1), which balances the competition between adsorption/desorption rate-limiting steps [41,42], is here expected at a moderate *T*_C_ (Table S4). Thus, increasing *T*_C_ up to 800 °C results in a pure-single phase SP and enhanced occupancy of 16*d* sites by Cr^3+^, Mn^3+^, and Ni^2+^ cations, but in non-optimal *e_g_* filling. Conversely, an occupancy of *e_g_* orbitals at octahedral sites closer to the OER-optimal value is achieved (at lower *T*_C_) but at the expense of oxide phase purity and 16*d* site occupancy by highly electrochemically active cations.

**Figure 3:**
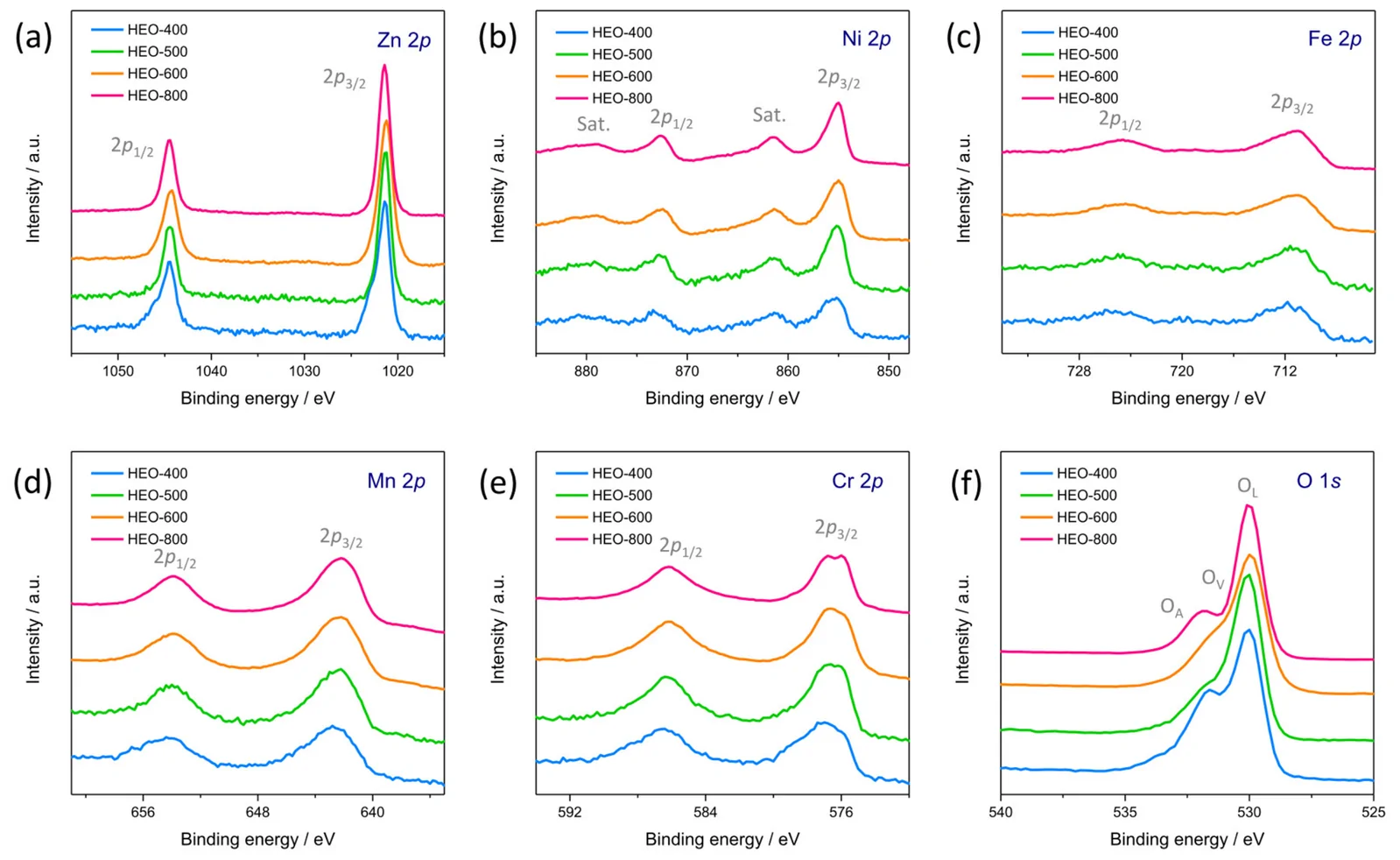
HRXPS spectra in the regions of (**a**) Zn 2p, (**b**) Ni 2p, (**c**) Fe 2p, (**d**) Mn 2p, (**e**) Cr 2p, and (**f**) O 1s core levels in oxides.

The species present on the surface of the electrocatalysts are investigated by XPS. All five TMs, oxygen, and adventitious carbon are detected in all the electrocatalysts (Figure S11). In the spectra of the reduced oxides, the decrease in the oxygen peak intensity compared to that of the pristine oxides confirms the reduction of the oxide surface (Figure S12). Figure 3 displays high-resolution X-ray photoelectron (HRXPS) spectra of the core levels in the oxides.

The binding energies (BEs) of the intense spin-orbit coupling components Zn 2*p*_3/2_ and Zn 2*p*_1/2_ (1021.4 and 1044.4 eV, respectively), detected in the Zn 2*p* region of the HRXPS spectra (Figure 3a), are characteristic of ZnO and indicate that zinc is present on the oxide surface as Zn(II) [11,34,43]. The shoulder on the higher-BE side of the two peaks in the spectrum of HEO-400 hints at the formation of zinc hydroxide [43]. The features detected in the Ni 2*p* region (Figure 3b)—specifically the two spin-orbit components at 855.0 and 872.6 eV and the satellites at 861.4 and 879.2 eV—reveal the presence of a single type of nickel species, namely Ni(II) in NiO and in mixed oxides [34,44]. The HRXPS spectra of the Fe 2*p* core level (Figure 3c), consisting of two broad peaks centered at BEs of 711.0 and 724.6 eV (corresponding to the Fe 2*p*_3/2_ and Fe 2*p*_1/2_ features, respectively), closely resemble the spectrum of Fe_3_O_4_ [45], suggesting the coexistence of Fe(III) (711.5 eV) and Fe(II) (710.1 eV) species [44], consistent with the results of other studies [34,46]. In the Mn 2*p* region (Figure 3d), two broad Mn 2*p*_3/2_ and Mn 2*p*_1/2_ peaks are detected at BEs (642.2 and 654.0 eV) intermediate between those typical of Mn (II) (641.6 and 653.3 eV) and Mn (III) (643.5 and 654.9 eV), which indicates the co-existence of Mn species in both oxidation states [11,47].

The peak of the Cr 2*p*_3/2_ core level (Figure 3e) consists of three contributions located at BEs of approximately 575.8, 576.8, and 578.7 eV; they reveal the presence of Cr(III) species in both oxide and hydroxide chemical environments and Cr(VI) species in oxide [48,49], as observed also in previous studies [15,34]. Finally, in the O 1*s* region (Figure 3f), three contributions are resolved at BEs of ca. 530.0, 531.5, and 533.2 eV; they are commonly attributed to lattice oxygen (O_L_), “oxygen vacancies” (O_V_), originating from surface O-anions adjacent to lattice vacancies, passivated with adsorbed hydrogen or surface OH^−^ formed upon adsorbed water [50,51], and loosely bound water (O_A_) [51]. It is worthwhile noticing that “oxygen vacancies” may occur in different electronic configurations and charge states, which can induce distinct local structural and electronic perturbations [52,53]. The present XPS analysis does not allow these individual vacancy states to be unambiguously distinguished. Therefore, the O_V_ contribution is used here as a phenomenological descriptor of oxygen-defect-related surface species. Their concentration varies with *T*_C_ (Figure S13b) and the O_V_/O_L_ ratio decreases with increasing *T*_C_ (Figure S13c).

Regarding the reduced oxides (Figure S14), no significant change in peak position, width, or relative intensity is observed in the HRXPS spectra of HEO-400/R compared to those of HEO-400, consistent with the results of Rietveld analysis. In the case of HEO-600/R, the BE positions of Ni, Fe, and Cr 2*p* core levels do not appreciably vary with respect to those of HEO-600, although the peaks broaden. Conversely, additional peaks located at higher BEs are clearly visible in the Zn, Mn 2*p*, and O 1*s* regions, suggesting a distribution of oxidation states and local coordination environments. For the remaining samples, all spectra appear to consist of a higher-intensity (in most cases broadened) replica of the original profile superimposed on the weakened original signal, reflecting the multi-phasic nature of the material obtained upon reduction.

### 3.2. Electrochemical Characterization

For each catalyst formulation, multiple independently prepared “symmetric” MEAs were fabricated and tested to verify the reproducibility of the observed electrochemical trends. The four “symmetric” assemblies fabricated by pairing electrodes derived from the same catalyst material, employing its oxidized form (HEO-*T*_C_) as the anode and its reduced counterpart (HEO-*T*_C_/R) as the cathode, were labeled MEA 1–4 in order of increasing calcination temperature. The results shown through this section are representative of their measured behavior. Following cell assembly, a short galvanostatic conditioning step at low current density was performed to promote electrode activation (Figure S15). After an initial transient, the cell voltage stabilized for all assemblies except MEA 4. The deterioration observed for this MEA may be partially associated with the Zn leaching occurring during the reduction treatment of HEO-800 (Figure S4h), although this effect cannot be conclusively established.

**Figure 4:**
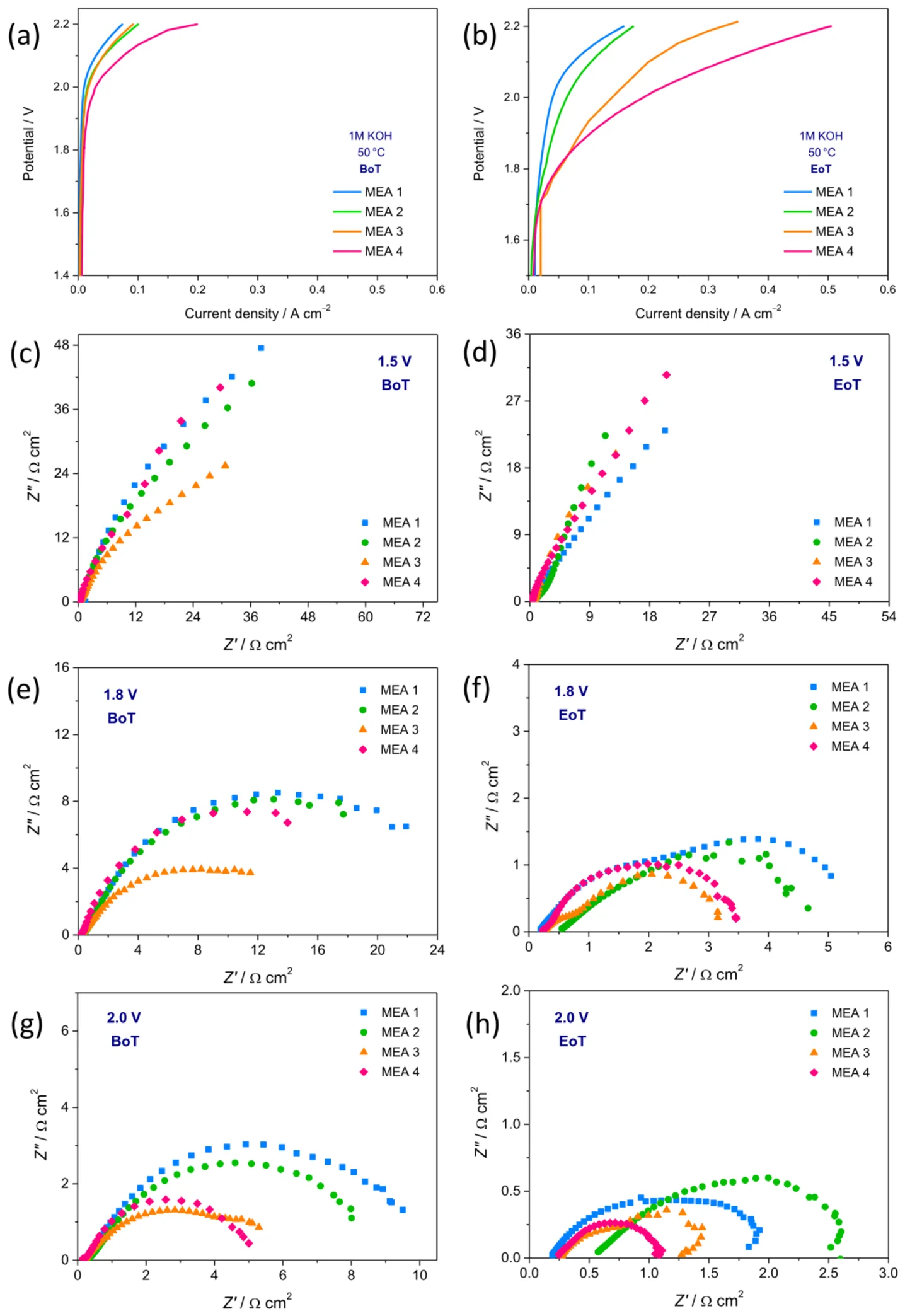
(**a**,**b**) AEMWE cell polarization curves and (**c**–**h**) Nyquist plots recorded at 50 °C at (**c**,**d**) 1.5 V, (**e**,**f**) 1.8 V, and (**g**,**h**) 2.0 V cell potential at the (**a**,**c**,**e**,**g**) beginning of test (BoT) and (**b**,**d**,**f**,**h**) end of test (EoT).

The initial electrochemical performance of the MEAs was assessed through beginning-of-test (BoT) polarization curves and EIS measurements to determine the maximum achievable current density (*J*_C_), series resistance (*R*_S_), and polarization resistance (*R*_P_). Following a galvanostatic chrono-potentiometric durability test, the same analyses were repeated at the end of the test (EoT). The corresponding polarization curves and impedance spectra are reported in Figure 4. The electrochemical performance, as monitored by *J*_C_ (Figure 4b), improves as *T*_C_ increases and O_V_-concentration decreases, consistent with the enhanced OER activity observed for the (Cr,Mn,Fe,Co,Ni)-HEO electrocatalyst in a previous study [39] and the literature reports on the effect of changes in coordinatively unsaturated metal cations [54], respectively.

It is worth noting that the maximum current density achieved by the Co-free HEO-based MEAs is significantly lower than that previously reported for “first-generation” (Cr,Mn,Fe,Co,Ni)-HEO MEAs synthesized under optimized calcination conditions (0.51 vs. 1.33 A cm^−2^ at V_C_). Nevertheless, when the comparison is restricted to electrodes calcined under identical conditions (Table S5), the “second-generation” HEO-800||HEO-800/R MEA exhibits a slightly superior performance, delivering 0.47 A cm^−2^ at V_C_ compared with its first-generation counterpart [15].

**Figure 5:**
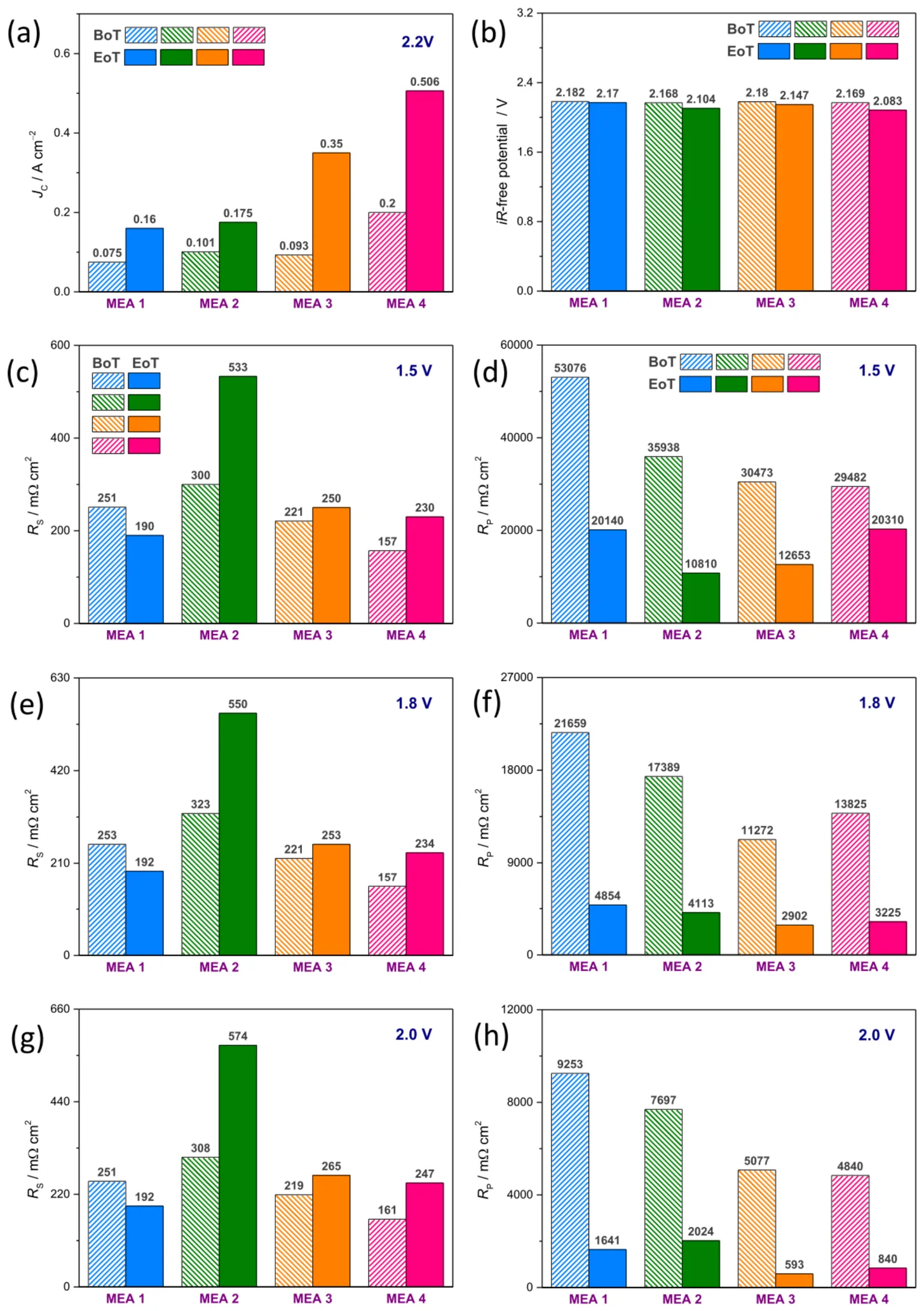
(**a**) Current density (JC) achieved at the cut-off (VC = 2.2 V) and (**b**) *iR*-free potential. (**c**,**e**,**g**) Series resistance (RS) and (**d**,**f**,**h**) polarization resistance (RP) at (**c**,**d**) 1.5 V, (**e**,**f**) 1.8 V, and (**g**,**h**) 2.0 V.

As commonly reported in the literature [55], the *R*_S_ vales were determined from the high-frequency intercept of the Nyquist plot with the real axis, whereas the *R*_P_ vales were calculated as the difference between the low- and high-frequency intercepts on the same axis. The evolution of *J*_C_, the *iR*-free potential (*V*_0_), *R*_S_, and *R*_P_ at different applied voltages is summarized in Figure 5. Overall, all MEAs exhibit an increase in *J*_C_ after the durability test, accompanied by a decrease in both *V*_0_ and *R*_P_, suggesting an improvement in electrochemical performance following operation. In contrast, a reduction in *R*_s_ is observed only for MEA 1.

**Figure 6:**
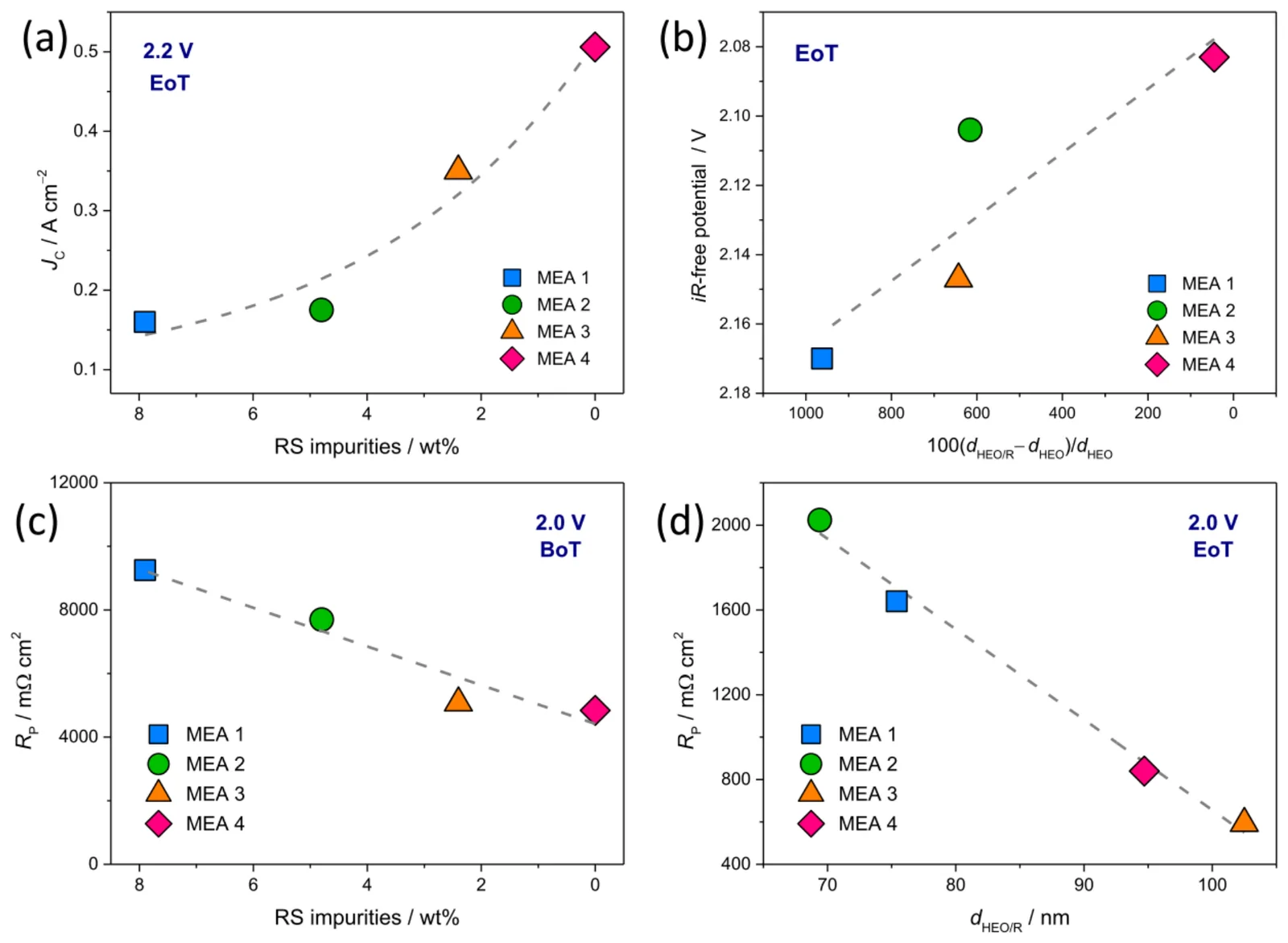
Dependence of (**a**) JC and (**b**) *iR*-free potential at the EoT on the amount of RS impurities and the relative increase in the average size of the crystallites in reduced oxides, respectively. Dependence of RP at 2.0 V (**c**) at the BoT and (**d**) EoT on the amount of RS impurities and average size of crystallites in reduced oxides (dHEO/R).

Understanding more deeply how the physicochemical properties of the pristine electrode materials influence electrochemical performance is of crucial importance to control their synthesis and tailor them to desired specifications. In the present case, a comparison of the physicochemical properties of the material and the electrochemical behavior of the MEAs (Figure 6), reveals that the purity degree of the oxide acting as the anodic catalyst strongly influences both the maximum current density achievable at the EoT and the polarization resistance at the BoT. The fewer the RS impurities, the higher the *J*_C_ value (Figure 6a) and the lower the *R*_P_ value (Figure 6c). This finding is consistent with the commonly recognized role of oxide phase purity on performance in applications such as catalysis, electrocatalysis, photocatalysis, and electronics [9,15,56,57,58,59]. Of course, the enhanced occupancy of 16*d* sites by catalytically active Cr^3+^, Mn^3+^, and Ni^2+^ cations, achieved concurrently at higher *T*_C_ (Figure S10b), also contributes to improve *J*_C_ and reduce *R*_P_ enhancing the OER performance.

The properties of the reduced oxide acting as the cathodic catalyst seem to mainly affect both the *iR*-free potential and the polarization resistance at the EoT. The lower the sintering effects during the oxide reduction process (the larger the surface available for reaction), the lower the *V*_0_ value (Figure 6b). Conversely, a larger average crystal size in the reduced oxide yields a lower *R*_P_ value at the EoT (Figure 6d). This behavior might be attributed to a decrease in the agglomeration degree of H_2_-reduced oxide grains that accompanies the crystal size growth, which may induce an improvement in the HER performance [60].

The picture that emerges from the results presented above is that the physicochemical properties of the electrode materials obtained by varying *T*_C_ and *T*_R_ can exert both synergistic and antagonistic effects on the electrochemical performance of the MEAs fabricated with them.

### 3.3. Long-Term Durability

**Figure 7:**
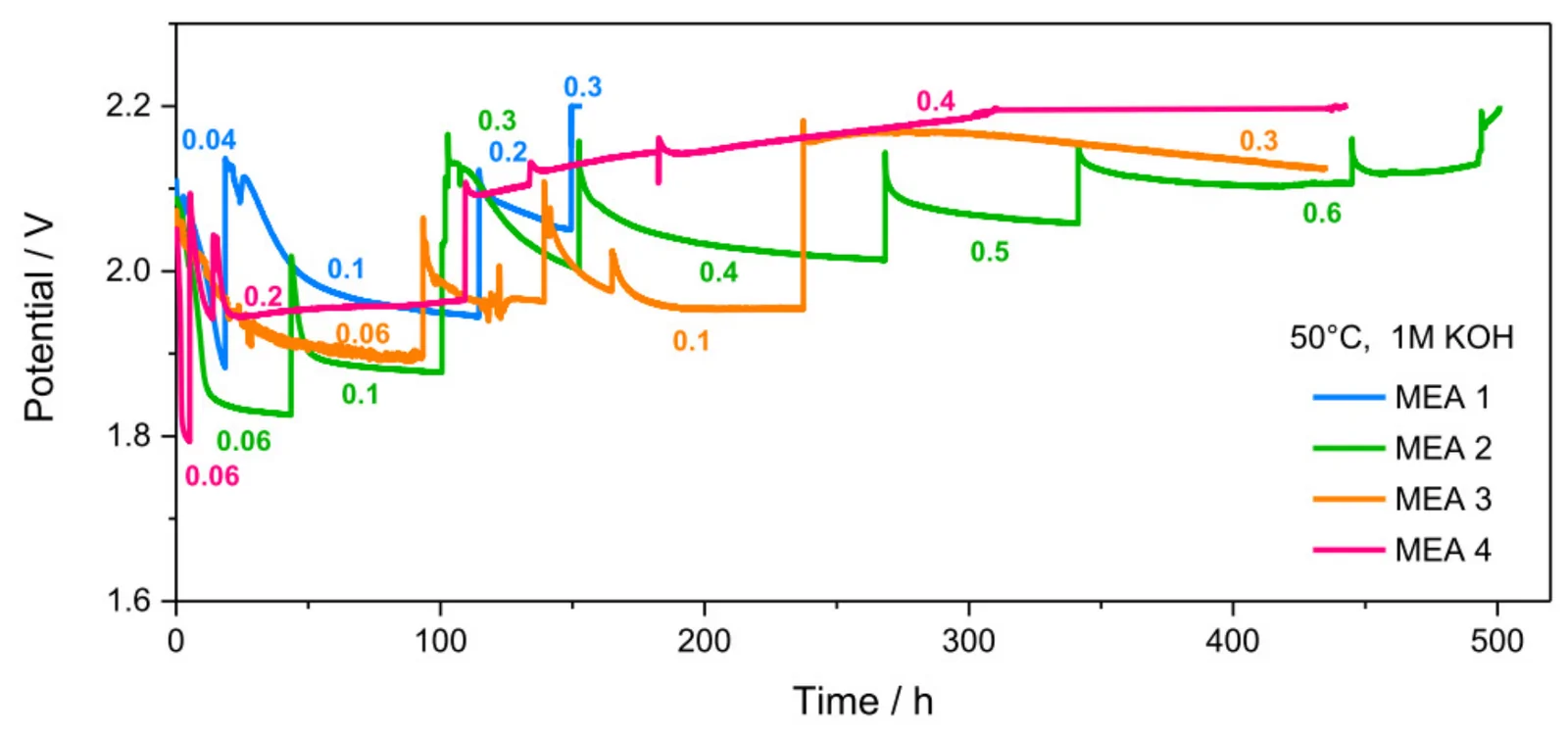
Durability tests at ambient pressure (the current density values, expressed in A cm^−2^, are indicated).

Long-term durability tests were performed under galvanostatic conditions to evaluate the operational stability of the Co-free HEO-based MEAs (Figure 7). The durability protocol was designed with progressive increases in current density to maintain the cell voltage below the imposed cut-off value (2.2 V) and thus avoid excessive degradation of the cell components. Consequently, the voltage evolution cannot be directly translated into a conventional degradation rate, which would require constant-current operation. During the initial stage of operation, all assemblies exhibited a conditioning period characterized by voltage fluctuations and progressive stabilization of the cell potential. This behavior is commonly associated with electrode wetting, electrolyte redistribution within the porous catalyst layers, ionomer hydration, and gradual improvement of the electrode-membrane interfacial contact. Following activation, MEA 1, MEA 2, and MEA 3 displayed remarkably stable operation over several hundred hours despite the progressive increase in the applied current density. In particular, MEA 3 maintained stable performance for more than 450 h, while MEA 2 operated for approximately 500 h and sustained current densities up to 0.6 A cm^−2^. Although no detailed post-mortem structural or chemical analyses were performed, the ability of these assemblies to preserve a nearly constant cell voltage under increasingly demanding operating conditions demonstrates the robustness of the Co-free HEO-based electrodes and suggests that no severe degradation phenomena occurred within the electrode layers or at the electrode-membrane interfaces during long-term operation. Furthermore, the stable series resistance observed during long-term operation suggests that the FAA3-50 membrane and FAA-3 ionomer maintained adequate ionic conductivity under the adopted operating conditions, with no evidence of severe degradation during the test period. The superior durability observed for MEA 2 and MEA 3 can be related to the more favorable balance between phase purity, crystallinity, and microstructural properties of the corresponding HEO catalysts. As discussed above, intermediate calcination temperatures promote the formation of highly homogeneous spinel structures while limiting excessive particle growth, thereby ensuring an adequate density of catalytically active sites together with sufficient structural stability under alkaline electrolysis conditions. In contrast, MEA 4 exhibited a progressive increase in cell voltage and the poorest long-term stability. This behavior is consistent with the physicochemical characterization of HEO-800/R, which revealed significant Zn depletion after the reduction treatment. The loss of Zn may induce local compositional heterogeneities and structural modifications that negatively affect catalyst stability and electrode integrity during prolonged operation. Although the exact degradation mechanism cannot be unequivocally identified based on the available data, the results suggest that excessive thermal treatment can compromise the long-term stability of the catalyst despite improving oxide phase purity.

From a broader perspective, the durability results highlight that catalyst optimization for AEMWE cannot be based solely on maximizing initial electrochemical activity. Instead, a careful balance between phase purity, microstructural stability, and compositional robustness is required to achieve sustained long-term performance. The ability of the best-performing Co-free HEO-based MEAs to operate stably for hundreds of hours demonstrates the potential of Co-free high-entropy oxides as sustainable electrode materials for future AEMWE systems and confirms the viability of the proposed electrode design strategy beyond short-term electrochemical evaluations.

## 4. Concluding Remarks

This study establishes relevant correlations between the physicochemical properties of pristine electrode materials and the electrochemical performance of the corresponding MEAs, providing valuable insights for the rational control of synthesis parameters and the targeted optimization of HEO-based electrocatalysts. In particular, the phase purity of the oxide employed as the anodic catalyst was found to correlate with the maximum current density and polarization resistance of the corresponding MEAs. The properties of the reduced oxide were observed to correlate with the polarization resistance and *iR*-free potential evolution during long-term operation; however, the individual contribution of the different physicochemical parameters could not be independently assessed. Although the MEAs exhibited stable electrochemical performance over several hundred hours, the present results do not allow definitive conclusions regarding possible catalyst dissolution, oxidation-state evolution, electrode restructuring, or membrane degradation. Dedicated post-mortem characterization (XRD, XPS, electron microscopy, and electrolyte analysis) will be required to elucidate the underlying degradation mechanisms. Furthermore, the results demonstrate that the electrode design strategy previously developed for “first-generation” (Cr,Mn,Fe,Co,Ni)-HEO-based MEAs, capable of achieving performances approaching industrially relevant levels, can be successfully transferred to “second-generation” cobalt-free HEOs (Cr,Mn,Fe,Ni,Zn). However, the removal of cobalt, while significantly improving the sustainability profile of the materials by eliminating a critical raw material, inevitably leads to a reduction in overall electrochemical performance. This finding highlights the need to carefully balance catalytic activity, material availability, and long-term stability in the development of next-generation electrocatalysts for AEMWE. This work demonstrates that fine tuning of synthesis conditions represents an effective strategy to optimize the physicochemical properties of Co-free HEO electrodes, enabling the design of more sustainable and scalable electrocatalytic materials. Although the observed trends provide useful guidelines for catalyst design, additional studies involving a larger number of compositions and the independent control of specific physicochemical parameters will be required to establish definitive structure–property relationships. These results contribute to the development of environmentally and socio-economically responsible solutions for the future implementation of green hydrogen production technologies.

## Data Availability

The original contributions presented in this study are included in the article/Supplementary Materials. Further inquiries can be directed to the corresponding authors.

## Supplementary Materials

The following supporting information can be downloaded at: https://www.mdpi.com/article/10.3390/nano16161034/s1, Figure S1: schematic description of the synthesis route; Figure S2: results of TPR analysis carried out in a H_2_/Ar atmosphere for 30 min; Figure S3: (a) airbrush system for electrodeposition of electrocatalytic inks, (b) anode (~2.5 mg cm^−2^ loading), (c) cathode (3 mg cm^−2^ loading), (d) Fu-matech® membrane, (e) membrane electrode assembly (MEA, 5 cm^2^ active area), (f) exploded scheme and (g) picture of zero-gap cell for anion exchange membrane water electrolysis (AEMWE); Figure S4: results of SEM/EDX analyses on (a–d) OER electrocatalysts (e–h) HER electrocatalysts. Pie-plots show the relative percentage of each metal in the combination; Table S1: relative percentage of each metal (%) in the as-calcined and reduced oxides, as determined via SEM-EDX analysis; Figure S5: SEM/EDX elemental map of sample HEO-600; Figure S6: Rietveld refinements for OER electrocatalysts; Table S2: results of Rietveld refinements for OER electrocatalysts; Figure S7: Rietveld refinements for HER electrocatalysts; Table S3: results of Rietveld refinements for HER electrocatalysts; Figure S8: dependence (a) of the amount of rock-salt (RS) impurities and (b) average size of the crystallites (*d*_HEO_) in oxides on calcination temperature (*T*_C_) and (c) of relative increase in the average size of the crystallites in reduced oxides (*d*_HEO/R_) on the relative distance of reduction temperature (*T*_R_) from *T*_C_; Figure S9: MRS spectra of OER electrocatalysts; Figure S10: limiting situations of (a) completely random distribution of cations (CRCD) and (b) equilibrium distribution (ECD), to which (Cr,Mn,Fe,Ni,Zn) HEOs calcined at low and high temperature ideally tend, respectively; Table S4: configurational entropy $∆S_{config}$ and occupancy of *e_g_* orbitals at octahedral sites for the CRCD (Figure S10a) and ECD (Figure S10b); Figure S11: survey XPS spectra of the (a) oxides and (b) reduced oxides; Figure S12: comparison between survey spectra of an oxides and a reduced oxide; Figure S13: (a) fits of XPS spectra in the regions of O 1s core levels. (b) Concentration of surface O-species: lattice oxygen (O_L_), oxygen vacancies (O_V_) and loosely bound water (O_A_). (c) O_V_ to O_L_ ratio; Figure S14: HRXPS spectra in the regions of (a) Zn 2p, (b) Ni 2p, (c) Fe 2p, (d) Mn 2p, (e) Cr 2p and (f) O 1s core levels in reduced oxides; Figure S15: conditioning (for ca. 2 h), aimed at activating the MEAs; Table S5: main parameters summarizing catalyst composition, operating conditions, catalyst loading, cell performance, and durability metrics of the Co-containing (previous work) and Co-free (present work) HEO-based electrocatalysts; Figure S16: (a,b) AEMWE cell polarization curves after iR-correction. (c) General improvement of the cell performance after operation as monitored by the decrease of the values of *iR*-free potential as a function of those of the maximum current density. References [15,38,39,61,62,63,64,65] are cited in Supplementary Materials.
