# Supplementary material for: (Cr,Mn,Fe,Ni,Zn) High-Entropy Oxides as Electrocatalysts for Green Hydrogen Production via Anion Exchange Membrane Water Electrolysis
Source: Nanomaterials (Basel). 2026 Aug 20;16(16):1034. doi: 10.3390/nano16161034 (PMC13516241; doi:10.3390/nano16161034)
Supplement: Supplementary file 1 [file nanomaterials-16-01034-s001.zip › nanomaterials-4497359-supplementary.pdf]

Supporting Information

(Cr,Mn,Fe,Ni,Zn) High-Entropy Oxides as Electrocatalysts for Green Hydrogen Production via Anion Exchange Membrane Water Electrolysis

Sabrina Campagna Zignani [1,*], Marta Fazio [1], Mariarosaria Pascale [1], Chiara Alessandrello [2], Claudia Triolo [2,3], Maria Grazia Musolino [2,3,*] and Saveria Santangelo [2,3,*]

[1] Institute of Advanced Technologies for Energy (ITAE), the National Research Council (CNR), Salita S. Lucia sopra Contesse 5, 98126 Messina, Italy; fazio@itae.cnr.it (M.F.); pascale@itae.cnr.it (M.P.)

[2] Department of Civil, Energy, Environmental and Materials Engineering (DICEAM), Mediterranean University of Reggio Calabria, Via R. Zehender, Loc. Feo di Vito, 89122 Reggio Calabria, Italy; c.alessandrello@unirc.it (C.A.); claudia.triolo@unirc.it (C.T.)

[3] National Reference Center for Electrochemical Energy Storage (GISEL), National Interuniversity Consortium for the Science and Technology of Materials (INSTM), 50121 Florence, Italy

* Correspondence: sabrina.zignani@itae.cnr.it (S.C.Z.); mariagrazia.musolino@unirc.it (M.G.M.); saveria.santangelo@unirc.it (S.S.)

Synthesis of electrocatalysts and preparation of membrane electrode assemblies

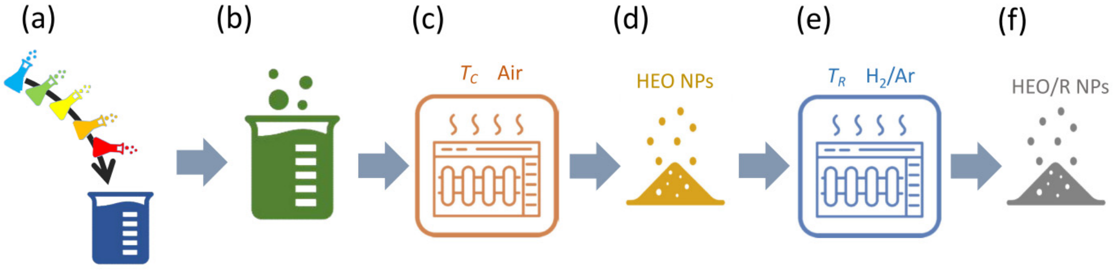

Figure S1. Schematic description of the synthesis route. (a,b) Precursor solution preparation, (c) gel calcination, (d) as-obtained high-entropy oxide (HEO) nanoparticles (NPs) to be used as electrocatalysts at the cell anode, (e) reduction of HEO NPs in H_2/Ar-atmosphere and (f) as-obtained reduced high-entropy oxide (HEO/R) NPs to be used as electrocatalysts the cell cathode.

HEOs were prepared using chromium (III) acetate hydroxide (purity >98%, CAS No. 39430-51-8), manganese(II) acetate tetrahydrate (purity >99%, CAS No. 6156-78-1), iron acetate (purity >95%, CAS No. 3094-87-9), nickel (II) acetate tetrahydrate (purity >98%, CAS No. 6018-89-9) and zinc(II) acetate dihydrate (purity >98%, CAS 5970-45-6) as Cr, Mn, Fe, Ni and Zn sources, respectively. Stoichiometric amounts of these salts were dissolved in 60 ml water, one at a time (Figure S1a), to obtain a 0.36M solution. After magnetic stirring at 250 rpm for 30 min, monohydrate citric acid (purity 98%; CAS No. 5949-29-1) was added as a complexing agent (1:1.5 total metal: citric acid ratio). The resulting mixture (Figure S1b) was further stirred at 350 rpm for 2h at 70°C to form a gel. After drying at 80°C overnight, the product was calcined in static air in a muffle furnace (Figure S1c) to remove organics and obtain multimetallic oxides to be used as OER electrocatalysts for the cell anode. The anodic materials were coded as HEO-T_C, with T_C (400, 500, 600 or 800 °C) denoting calcination temperature. Temperature was increased at a rate of 10 °C min^{-1} and kept constant for 2 h at the selected value. After thermal treatment, the material was rapidly cooled to room temperature (RT) outside the furnace. A portion of each oxide produced was utilised as catalyst for the OER at the anode (Figure S1d). The remaining

part was reduced under a 5% H_2/Ar flow to generate multimetallic phases to be used as cathodic material for the HER, hereafter coded as HEO-T_C/R. The reduction temperature of each material was determined by means of the temperature programmed reduction (TPR) analysis (Figure S2).

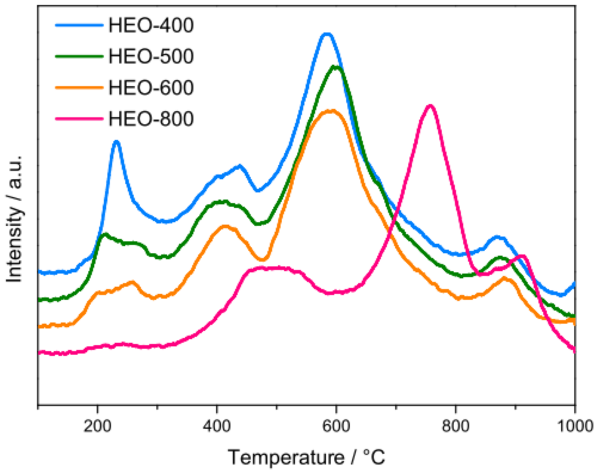

Figure S2. Results of TPR analysis carried out in a H_2/Ar atmosphere for 30 minutes.

The full reduction of HEOs in a 5% /Ar atmosphere and phase transformation to high-entropy alloys (HEAs) often requires temperatures from 750 °C to 900 °C [61], while partial reduction begins at lower temperatures (typically 400–600 °C). The reduction behavior is highly dependent on the composition, lattice structure and crystallization degree, as well as on the particle size. Nano-sized/highly-crystallized metal oxides often show lower/higher reduction temperatures compared to bulk /lower crystallinity materials.

In the TPR pattern of sample HEO-400, two major H_2 consumption peaks are detected at 220 and 600 °C; minor peaks are located at ca. 400 and 880 °C. As calcination temperature (T_C) increases, the peak at 220 °C progressively splits and decreases in intensity until vanishing at T_C = 800°C. For T_C < 800°C, the remaining peaks undergo no significant change, whereas at T_C = 800°C they shift at significantly higher temperatures (ca. 500, 750 and 930 °C), but without appreciable changes in their relative intensity. The lower-T H_2 consumption peaks can be ascribed to the reduction of Ni [62], while the higher-T ones can be associated to the multi-step reduction of higher valence metals [63].

Based on the above results, in order to ensure effective reduction of the most stable species, HEO-400, HEO-500, HEO-600, and HEO-800 were reduced at 990, 1020, 1040, and 1040 °C, respectively. The higher the reduction temperature of the sample compared to the temperature experienced during calcination, the greater the sintering effects are expected to be.

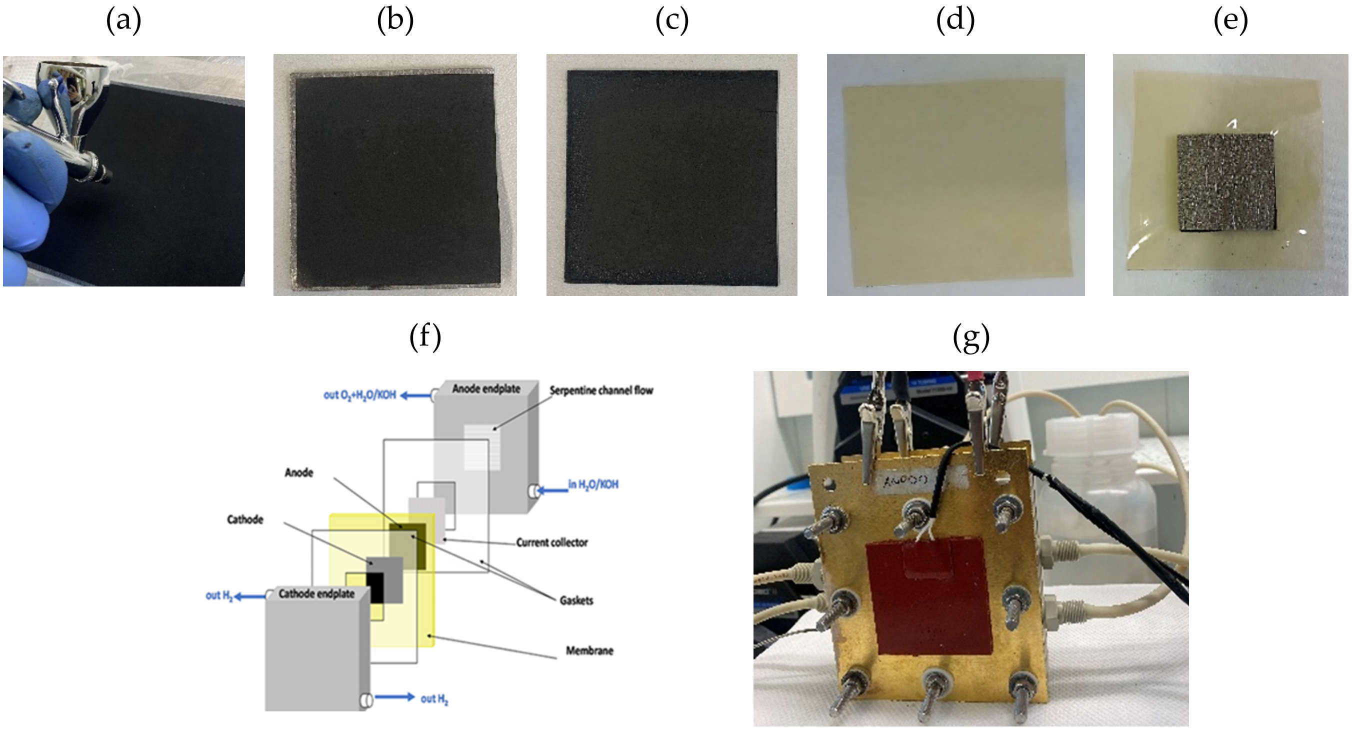

Figure S3. (a) Airbrush system for electrodeposition of electrocatalytic inks, (b) anode (~2.5 mg cm^{-2} loading), (c) cathode (3 mg cm^{-2} loading), (d) Fumatech® membrane, (e) membrane electrode assembly (MEA, 5 cm^2 active area), (f) exploded scheme and (g) picture of zero-gap cell for anion exchange membrane water electrolysis (AEMWE).

Physicochemical properties of electrocatalysts

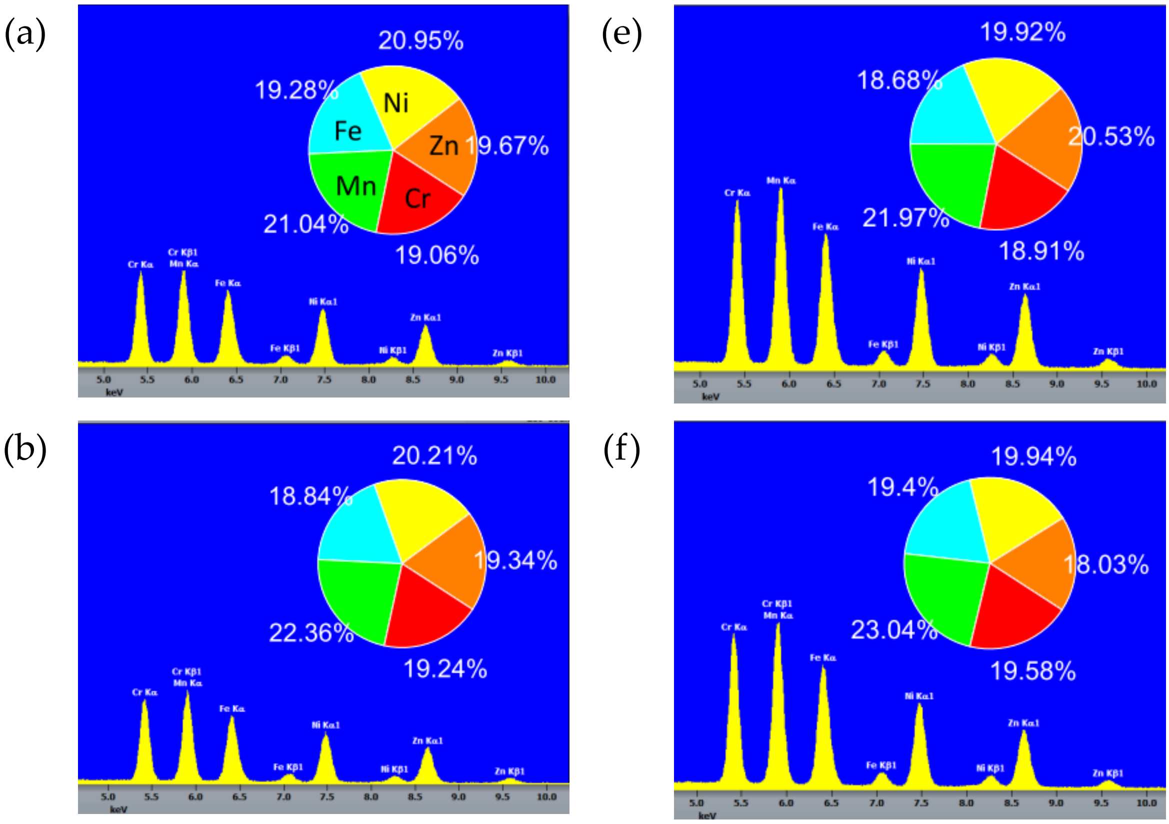

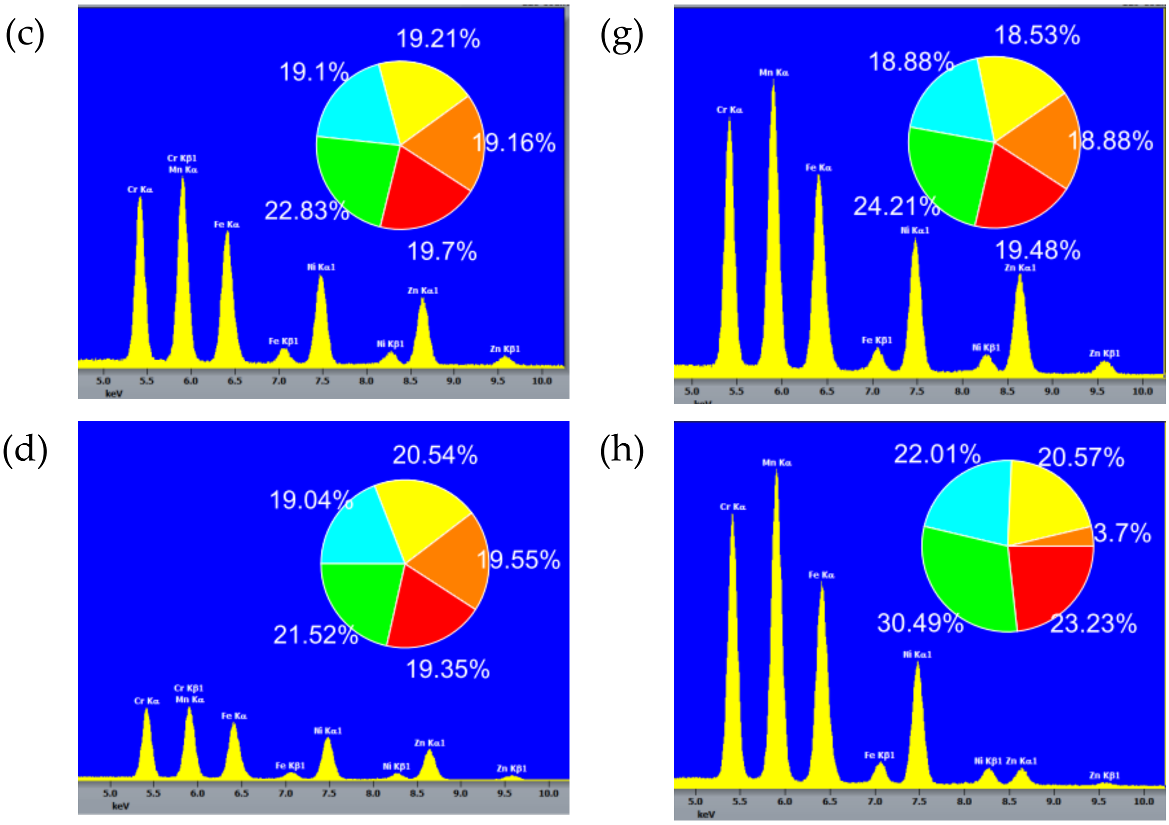

Figure S4. Results of SEM/EDX analyses on (a–d) OER electrocatalysts (a) HEO-400, (b) HEO-500, (c) HEO-600 and (d) HEO-800 (e–h) HER electrocatalysts (e) HEO-400/R, (f) HEO-500/R, (g) HEO-600/R and (h) HEO-800/R. Pie-plots show the relative percentage of each metal in the combination.

Table S1. Relative percentage of each metal (%) in the as-calcined and reduced oxides, as determined via SEM-EDX analysis.

**As-calcined oxides**	**Ni (%)**	**Fe (%)**	**Mn (%)**	**Cr (%)**	**Zn (%)**
HEO-400	20.95	19.28	21.04	19.06	19.67
HEO-500	20.21	18.84	22.36	19.24	19.34
HEO-600	19.21	19.10	22.83	19.70	19.16
HEO-800	20.54	19.04	21.52	19.35	19.55
**Reduced oxides**	**Ni (%)**	**Fe (%)**	**Mn (%)**	**Cr (%)**	**Zn (%)**
HEO-400/R	19.92	18.68	21.97	18.91	20.53
HEO-500/R	19.94	19.40	23.04	19.58	18.03
HEO-600/R	18.53	18.88	24.21	19.48	18.88
HEO-800/R	20.57	22.01	30.49	23.23	3.70

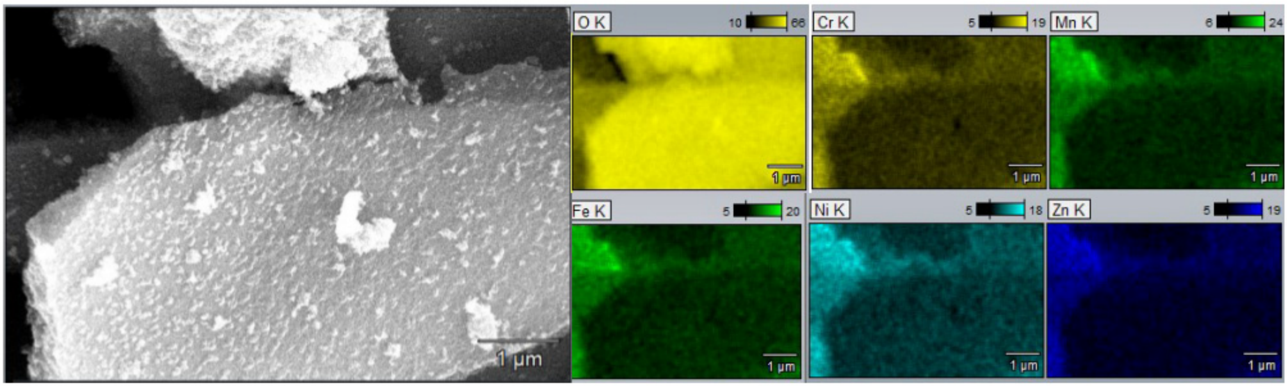

Figure S5. SEM/EDX elemental map of sample HEO-600.

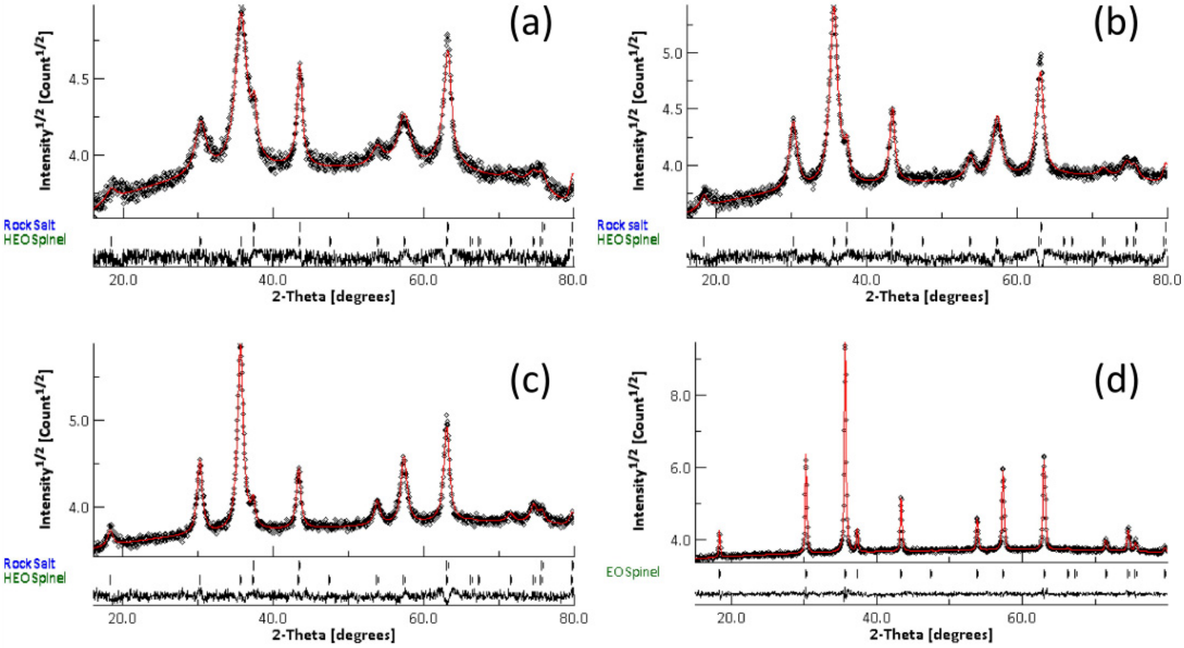

Figure S6. Rietveld refinements for OER electrocatalysts (a) HEO-400, (b) HEO-500, (c) HEO-600 and (d) HEO-800.

Table S2. Results of Rietveld refinements for OER electrocatalysts. SP and RS indicate spinel and rock-salt phases of the oxides, respectively. In the case of multiphase material, the average size of the oxide crystallites (d_{HEO}) is obtained as weighted average of present phases.

Sample	Phase	% w/w	Cell size / Å	Crystal size / nm	Microstrain	d_{HEO} / nm
HEO-400	SP	92.1	8.347±0.003	5.91±0.08	NS	7.1
	RS	7.9	4.157±0.001	21.2±2.1	0.0025±0.0004	
HEO-500	SP	95.2	8.512±0.0008	9.09±0.06	NS	9.7
	RS	4.8	4.1670±0.0008	22.3±1.6	0.00156±0.00006	
HEO-600	SP	97.6	8.3507±0.0005	13.3±0.1	0.0012±0.0004	13.8
	RS	2.4	4.168±0.001	32.4±2.0	0.0017±0.0005	
HEO-800	SP	100	8.34650±0.00006	65.3±0.1	0.00087±0.00002	65.3

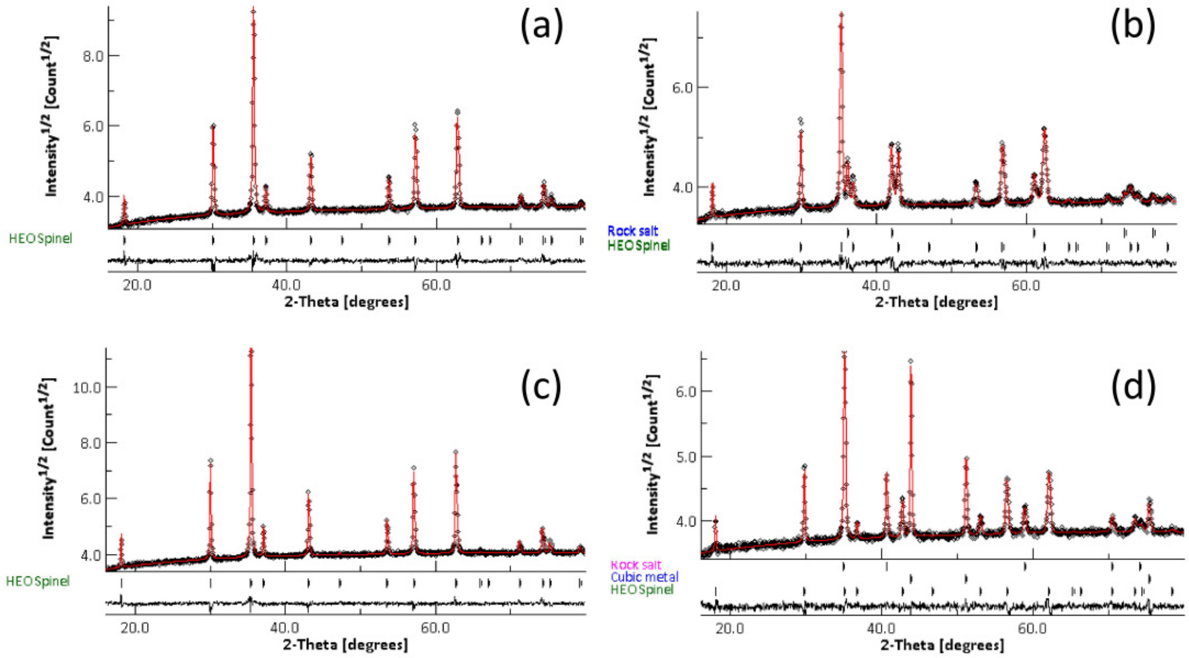

Figure S7. Rietveld refinements for HER electrocatalysts (a) HEO-400/R, (b) HEO-500/R, (c) HEO-600/R and (d) HEO-800/R.

Table S3. Results of Rietveld refinements for HER electrocatalysts. CM stands for cubic metal/alloy; SP and RS denote spinel and rock-salt phases of the compounds, respectively. The average size of the crystallites in the reduced material ($d_{HEO/R}$) is calculated as weighted average over the phases present.

Sample	Phase	% w/w	Cell size / Å	Crystal size / nm	Microstrain	$d_{HEO/R}$ / nm
HEO-400/R	SP	100	8.3706±0.0004	75.4±1.1	0.00100±0.00004	75.4
HEO-500/R	SP	73.4	8.4364±0.0002	81.4±3.4	0.00243±0.00006	69.4
	RS	26.6	4.3031±0.0002	35.8±0.9	NS	
HEO-600/R	SP	100	8.39662±0.00007	102.5±0.3	0.00057±0.00002	102.5
HEO-800/R	CM	28.6	3.57449±0.00007	118.9±8.3	0.00110±0.00007	94.7
	SP	53.7	8.4683±0.0003	75.2±3.3	0.00183±0.00008	
	RS	17.7	4.4403±0.0003	115.0±27.3	0.0018±0.0002	

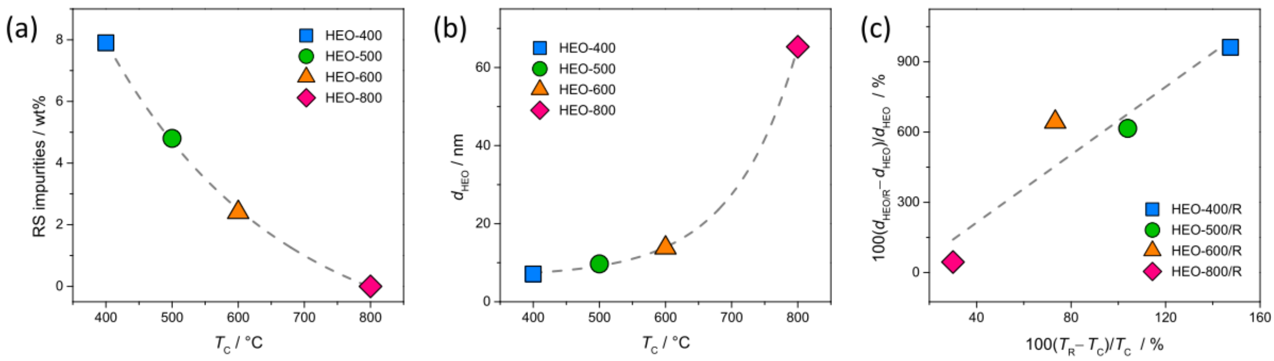

Figure S8. Dependence (a) of the amount of rock-salt (RS) impurities and (b) average size of the crystallites (d_{HEO}) in oxides on calcination temperature (T_C) and (c) of relative increase in the average size of the crystallites in reduced oxides ($d_{HEO/R}$) on the relative distance of reduction temperature (T_R) from T_C.

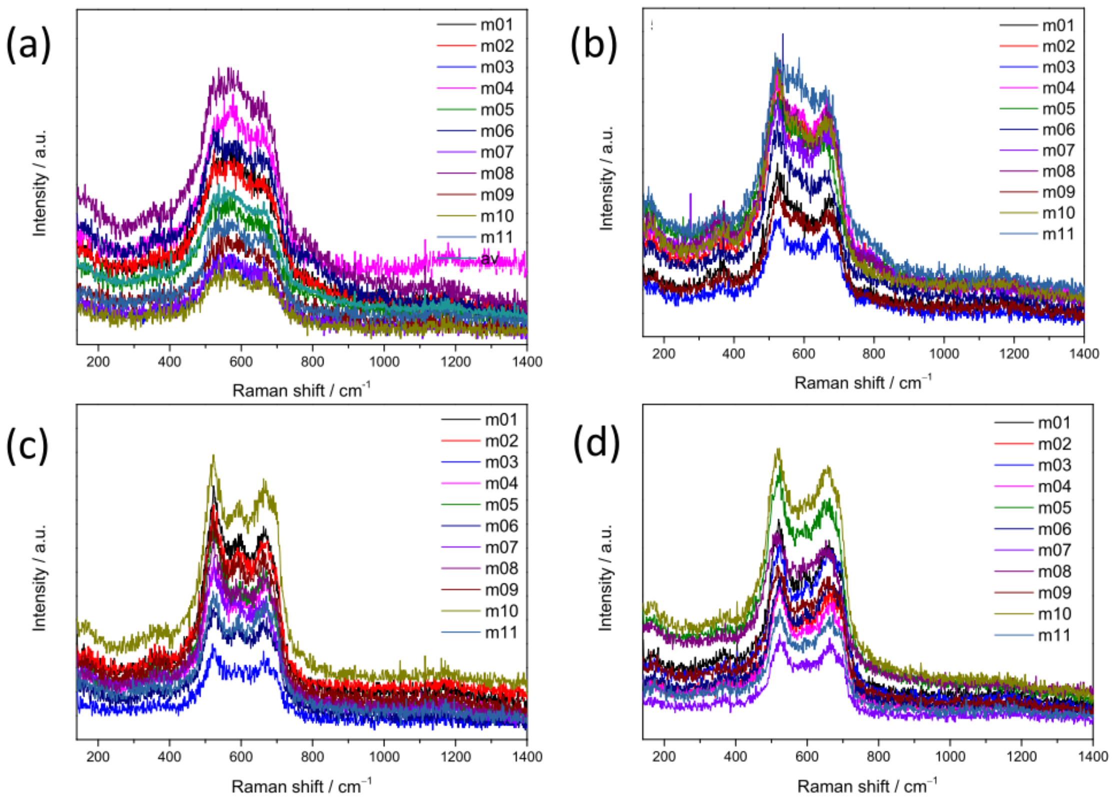

Figure S9. MRS spectra of OER electrocatalysts (a) HEO-400, (b) HEO-500, (c) HEO-600 and (d) HEO-800, measured at different random locations within each of them. The spectral difference observed at some locations in sample HEO-400 with the greatest amount of rock-salt (RS) impurities might be due to the presence of RS-nanodomains organized in larger clusters.

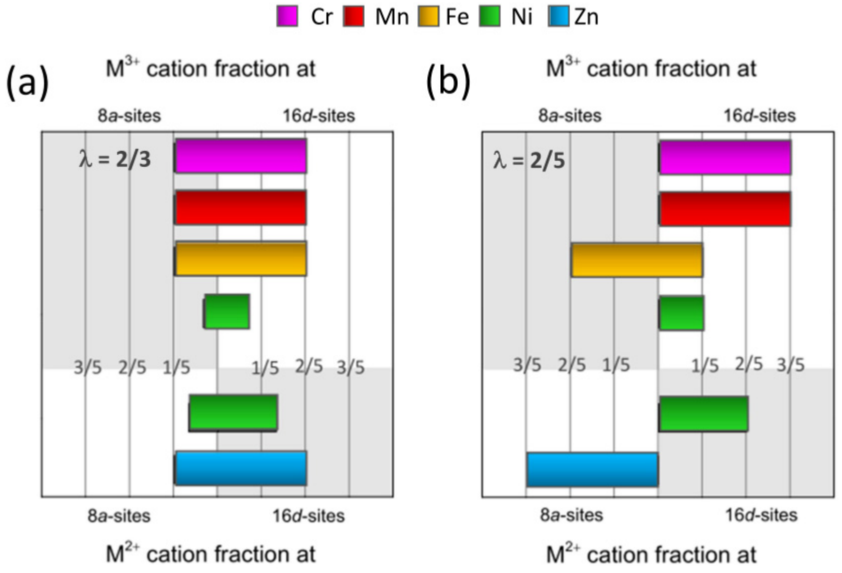

Figure S10. Limiting situations of (a) completely random distribution of cations (CRCD) [39] and (b) equilibrium distribution (ECD) [38], to which (Cr,Mn,Fe,Ni,Zn) HEOs calcined at low and high temperature *ideally* tend, respectively. λ denotes the inversion degree of the SP lattice, i.e. the fraction of M^{3+} cations occupying tetrahedral 8*a* sites in the $(M^{2+}_{1-\lambda}M^{3+}_{\lambda})_{8a}(M^{2+}_{\lambda}M^{3+}_{2-\lambda})_{16d}O_4$ lattice in place of octahedral 16*d* sites, unlike what happens in "normal spinels", $(M^{2+})_{8a}(M^{3+}_2)_{16d}O_4$; it decreases with increasing T_C.

The lower the calcination temperature (T_C), the greater the distance from thermodynamic equilibrium (owing to kinetic effects). In this limiting situation, the metal cations tend to distribute in the oxide lattice in a completely random manner, with the trivalent cations having a high octahedral 16*d* site preference energy also occupying the tetrahedral 8*a* sites [39]. Simple considerations on electronegativity lead to the distribution (a), where each cation is present in the 16*d* and 8*a* sub-lattices in a 2:1 ratio. On the contrary, the higher the calcination temperature, the closer the equilibrium is [38]. Hence, based on the optimization of the octahedral stabilization under the constraint of electroneutrality, in this opposite situation, the metal cations tend to distribute in the lattice as shown in scheme (b).

The configurational entropy of a multicomponent oxide with spinel structure can be roughly estimated as

$$\Delta S_{config} = -R\left[\left(\sum_{i=1}^{L} x_i ln x_i\right)_{8a\ \mathrm{sites}} + \left(\sum_{j=1}^{M} x_j ln x_j\right)_{16d\ \mathrm{sites}} + \left(\sum_{k=1}^{N} x_k ln x_k\right)_{\mathrm{O\ sites}}\right], \quad (1)$$

where R is the universal gas constant and x_i is the mole fraction of the ith species [40]. Neglecting the contribution from O-anion sub-lattice [64], based on the *ideal* occupation of the tetrahedral 8*a* sites and octahedral 16*d* sites depicted in Figure S10, equation (1) provides the values reported in Table S4.

Table S4. Configurational entropy ΔS_{config} and occupancy of e_g orbitals at octahedral sites [65] for the CRCD (Figure S10a) and ECD (Figure S10b).

CD	Site	Ideal occupation of the site	$\Delta S_{config,sub}$ / R *		ΔS_{config} / J K⁻¹ mol⁻¹	e_g **
CRCD	8*a*	$Cr^{3+}_{1/5}Mn^{3+}_{1/5}Fe^{3+}_{1/5}Ni^{3+}_{1/15}Ni^{2+}_{2/15}Zn^{2+}_{1/5}$	1.74 (1.61)	HE	43.32 (40.14)	3.6
	16*d*	$2(Cr^{3+}_{1/5}Mn^{3+}_{1/5}Fe^{3+}_{1/5}Ni^{3+}_{1/15}Ni^{2+}_{2/15}Zn^{2+}_{1/5})$	3.47 (3.22)	HE		
ECD	8*a*	$Fe^{3+}_{2/5}Zn^{2+}_{3/5}$	0.67 (0.67)	LE	30.62 (27.44)	2.2
	16*d*	$2(Cr^{3+}_{3/10}Mn^{3+}_{3/10}Fe^{3+}_{1/10}Ni^{3+}_{1/10}Ni^{2+}_{2/10})$	3.01 (2.63)	HE		

* The $\Delta S_{config,sub}$ values in brackets are obtained by considering the cations of the same element with different oxidation states as a single species. HE (> 1.5 R) and LE (< 1 R) stand for high-entropy and low-entropy, respectively.

** The optimal e_g filling of ≈ 3 (referred to the M_3O_4 formula unit) is here expected at a moderate T_C (400 < T_C < 800°C).

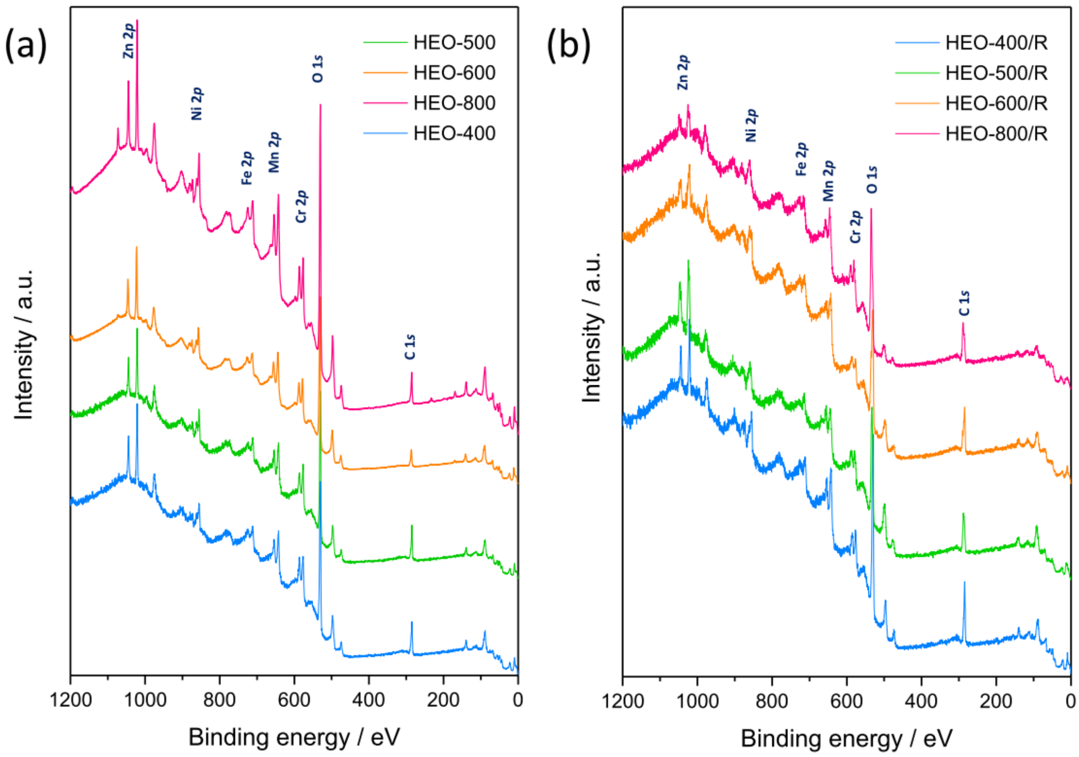

Figure S11. Survey XPS spectra of the (a) oxides and (b) reduced oxides.

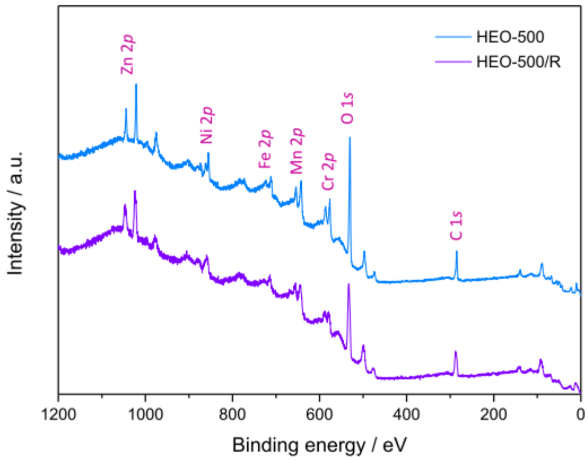

Figure S12. Comparison between survey spectra of an oxides and a reduced oxide.

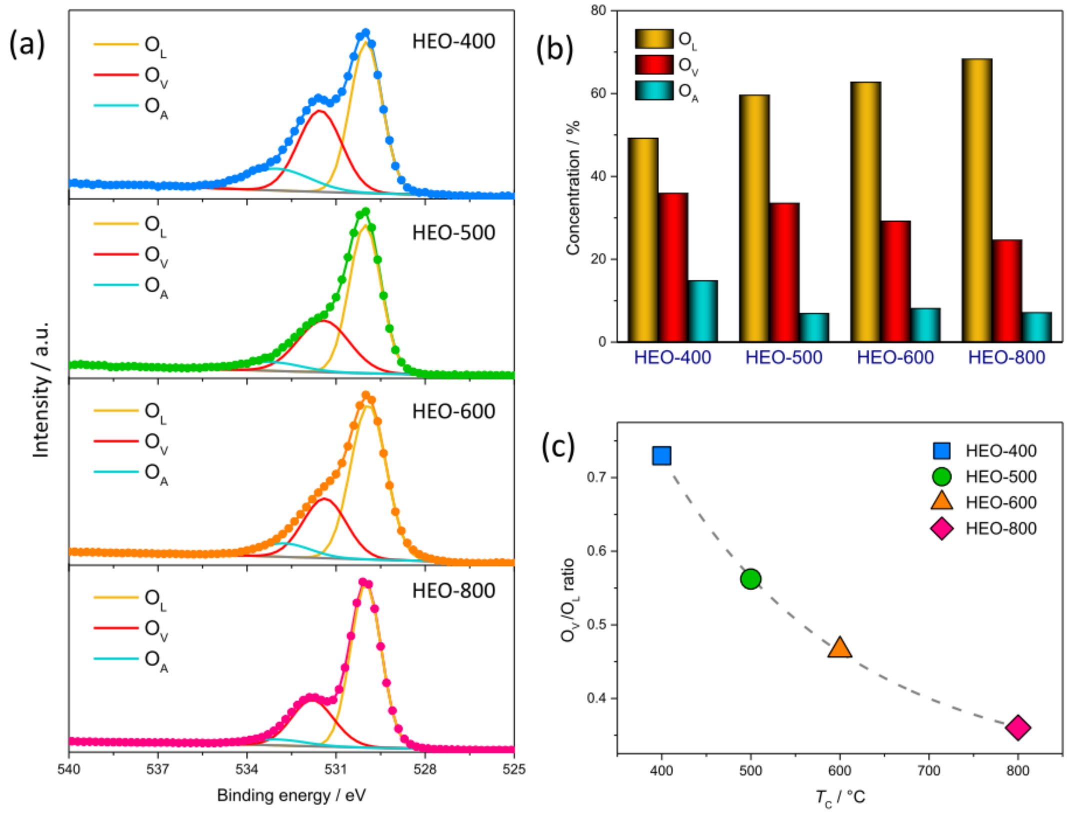

Figure S13. (a) Fits of XPS spectra in the regions of O 1*s* core levels. (b) Concentration of surface O-species: lattice oxygen (O_L), oxygen vacancies (O_V) and loosely bound water (O_A). (c) O_V to O_L ratio.

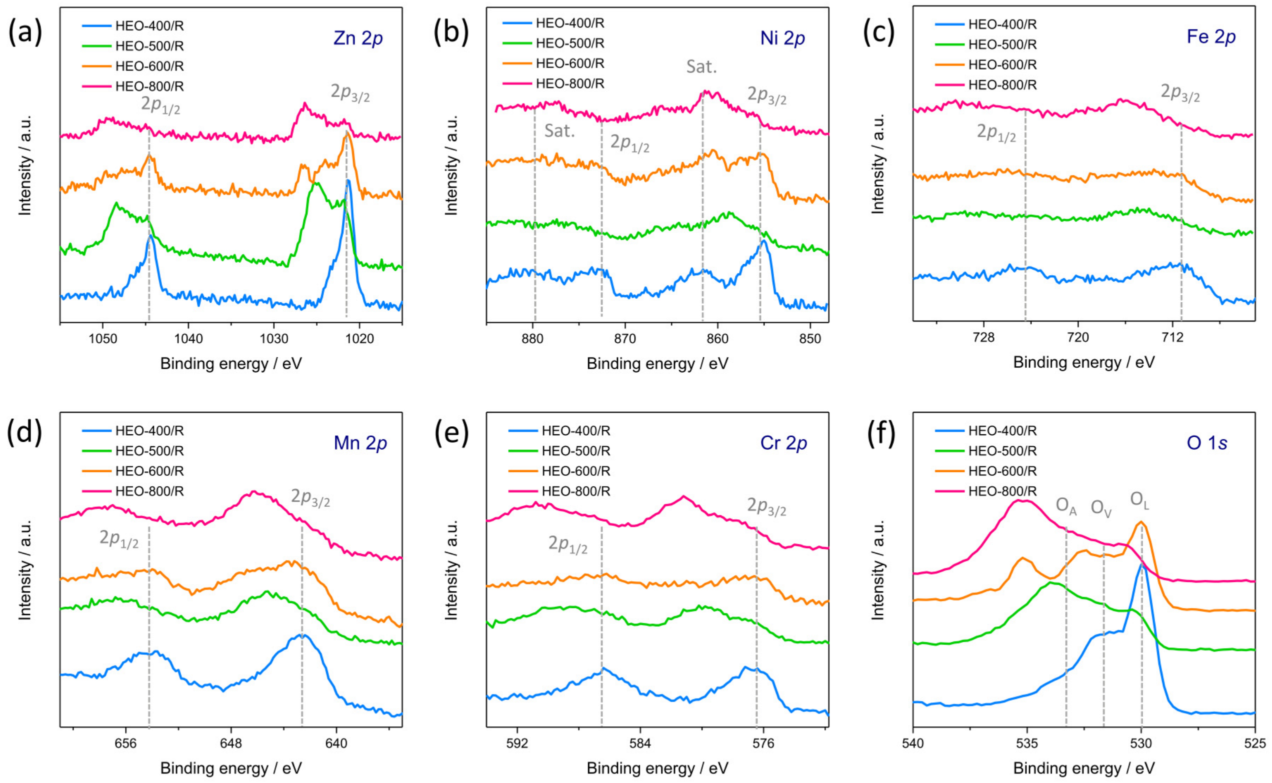

Figure S14. HRXPS spectra in the regions of (a) Zn 2*p*, (b) Ni 2*p*, (c) Fe 2*p*, (d) Mn 2*p*, (e) Cr 2*p* and (f) O 1*s* core levels in reduced oxides. Dashed lines indicate the BE positions of the spectral features in the pristine materials.

Electrochemical performance

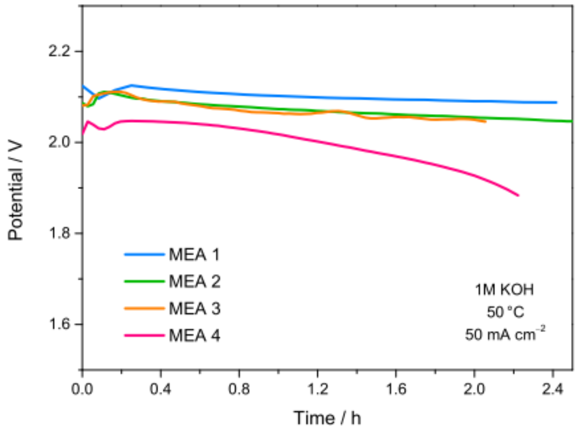

Figure S15. Conditioning (for ca. 2 h), aimed at activating the MEAs.

Table S5. Main parameters summarizing catalyst composition, operating conditions, catalyst loading, cell performance, and durability metrics of the Co-containing (previous work [15] and Co-free (present work) HEO-based electrocatalysts.

**Parameter**	**Co-containing HEO (previous work)**		**Co-free HEO (present work)**
	**Optimised configuration**		
Metal combination	Cr,Mn,Fe,Co,Ni	Cr,Mn,Fe,Co,Ni	Cr,Mn,Fe,Co,Ni
Calcination temperature	600 °C	800 °C	800 °C
Anode catalyst	HEO-600	HEO-800	HEO-800
Cathode catalyst	HEO-600/R	HEO-800/R	HEO-800/R
Catalyst loading (anode)	2.5 mg cm^{-}◎	2.5 mg cm^{-}◎	2.5 mg cm^{-}◎
Catalyst loading (cathode)	3 mg cm^{-}◎	3 mg cm^{-}◎	3 mg cm^{-}◎
Membrane	FAA3-50	FAA3-50	FAA3-50
Electrolyte	1M KOH	1M KOH	1M KOH
Temperature	50 °C	50 °C	50 °C
Active area	5 cm^2	5 cm^2	5 cm^2
Maximum current density at 2.2 V	1.33 A cm^{-2}	0.47 A cm^{-2}	0.51 A cm^{-2}
Durability	up to ~ 1100 h	up to ~ 140 h	up to ~ 500 h

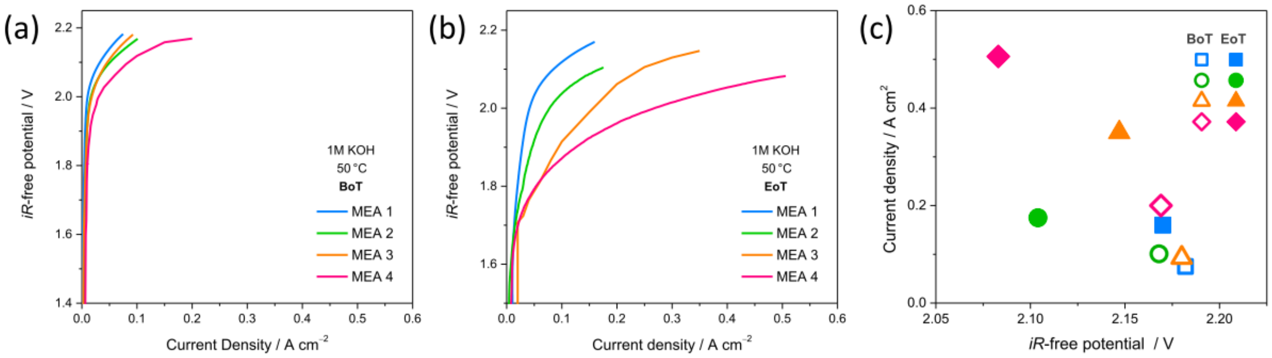

Figure S16. (a,b) AEMWE cell polarization curves after *iR*-correction. The shown data refer to the (a) beginning of test (BoT) and (b) end of test (EoT); for an easier comparison, the same scales are used for both plots. (c) General improvement of the cell performance after operation as monitored by the decrease of the values of *iR*-free potential as a function of those of the maximum current density.
